# Advances in deep learning for antimicrobial research

**DOI:** 10.3389/fmicb.2026.1934619

**Published:** 2026-09-22

**Authors:** Pan Mao, Chenrui Mao, Liu Zhang, Xin Li

**Affiliations:** 1 Department of Pharmacy, The Third Hospital of Changsha / The Affiliated Changsha Hospital of Hunan University, Hunan University, Changsha, Hunan, China; 2 Hunan Provincial Key Laboratory of Anti-Resistance Microbial Drugs, The Third Hospital of Changsha / The Affiliated Changsha Hospital of Hunan University, Hunan University, Changsha, Hunan, China; 3 School of Medicine, Hunan Normal University, Changsha, Hunan, China; 4 College of Information and Electrical Engineering, China Agricultural University, Beijing, China

**Keywords:** antimicrobial resistance, bioinformatics, deep learning, drug discovery, genomics

## Abstract

The rapid spread of antimicrobial resistance and the lag in the development of new antimicrobial drugs have become major challenges for global public health. Conventional methods for discovering antimicrobial drugs are characterized by long development cycles and high costs, which are further exacerbated by the rapid evolution of bacterial resistance. Therefore, there is an urgent need to develop efficient computational methods to accelerate the identification of drug resistance mechanisms and the research and development process of new antimicrobial drugs. In recent years, the new generation of artificial intelligence technologies represented by deep learning (DL) has made remarkable progress in antibacterial research. This paper reviews the current application status of DL in antimicrobial research, aiming to provide a comprehensive overview that covers background knowledge including technologies and principles, related case studies, and future perspectives. Firstly, the development of antibacterial drug discovery, the advancement of sequencing technique and basic concepts related to DL are introduced. Subsequently, a critical evaluation is conducted on the representative reports of DL in predicting drug resistance mechanisms and promoting antimicrobial drug discovery. Finally, the challenges faced by DL in antibacterial research and its future development directions are discussed, aiming to provide constructive suggestions and guidance for researchers in this field.

## 1 Introduction

Antimicrobial resistance (AMR) has been recognized by the World Health Organization as one of the top ten global public health threats, contributing to more than 1.27 million deaths annually worldwide (Okeke et al., 2024). Infections caused by drug-resistant bacteria pose a profound and escalating challenge to healthcare systems across the globe. Earlier projections estimated that AMR could cause up to 10 million deaths annually and result in a cumulative economic loss of USD 100 trillion by 2050 (O’Neill, 2016). Recent Global Burden of Disease estimates further indicate that AMR may account for 1.91 million attributable deaths and 8.22 million associated deaths annually by 2050 (Naghavi et al., 2024). The widespread use of antibiotics has significantly reduced mortality from infectious diseases; however, bacteria have continuously evolved to evade antimicrobial action through various mechanisms, including gene mutations, acquisition of resistance genes, modification of drug targets, and enhancement of efflux pump activity. These resistance mechanisms can arise from chromosomal mutations or be acquired via horizontal gene transfer (Baquero et al., 2021). Mobile genetic elements such as plasmids, transposons, and bacteriophages facilitate the rapid dissemination of resistance determinants across species boundaries (Khedkar et al., 2022). This has led to the emergence and global spread of so-called “superbugs,” including colistin- and carbapenem-resistant *Enterobacterale*, methicillin-resistant *Staphylococcus aureus* and multidrug-resistant *Mycobacterium tuberculosis* (MTB), severely limiting the effectiveness of existing treatment options (Gandhi et al., 2010; Vuong et al., 2016; Logan and Weinstein, 2017; Stewardson et al., 2019; Algammal et al., 2023). Conventional phenotypic assays are effective in determining microbial susceptibility to antibiotics, but they fail to elucidate the underlying genetic mechanisms of resistance and are incapable of predicting its evolutionary trajectory. Genome sequencing has provided a high-resolution tool for elucidating the mechanisms of AMR in pathogens. It enables comprehensive detection of genomic variations, facilitates the identification of resistance-associated genes, and reveals transmission dynamics and evolutionary patterns among microbial strains, thereby supporting both precision medicine and public health decision-making (Kayama et al., 2023; Wheeler et al., 2023; Djordjevic et al., 2024). However, the rapid advancement of sequence technique has also generated an unprecedented volume of genomic data, posing significant challenges to traditional bioinformatics methods in terms of processing efficiency and predictive accuracy. The application of artificial intelligence (AI), particularly deep learning (DL), has significantly advanced microbiomics and AMR research (Gao et al., 2024; Nguyen et al., 2024). DL models can automatically extract complex features from high-dimensional biological datasets, overcoming limitations associated with manually engineered features. DL has been widely applied to whole-genome sequencing data for identifying resistance-associated genes, predicting phenotypic resistance, and tracking transmission pathways of resistance determinants (Li Y. et al., 2021; Zhou et al., 2021; Jiang et al., 2022; Tang et al., 2023; Pei et al., 2024). Compared with traditional machine learning methods, DL provides improved representation learning and predictive capabilities for complex biological data (Nguyen et al., 2019; Khaledi et al., 2020; Van Camp et al., 2020). Moreover, DL approaches have demonstrated potential for discovering novel or cryptic resistance mechanisms (Li Y. et al., 2021; Pei et al., 2024). Beyond AMR prediction, DL has also shown considerable potential in antimicrobial drug discovery and design (Lluka and Stokes, 2023). By utilizing molecular graph representations and sequence-based information, DL models can generate compounds with desired chemical properties and biological activities, accelerating drug screening and optimization (Stokes et al., 2020). Furthermore, DL has been increasingly applied in protein structure prediction, target identification, and drug-target interaction modeling, facilitating more efficient antimicrobial development (Xia et al., 2023). Although several reviews have summarized DL applications in AMR prediction or antimicrobial drug development (Bilal et al., 2025; Melo et al., 2021; Cesaro et al., 2023), most have focused on either resistance analysis or drug discovery independently. Therefore, this review aims to provide a comprehensive overview of DL applications in antimicrobial research, including AMR prediction, genomic-based resistance analysis, antimicrobial discovery, and future challenges.

The remainder of this paper is structured as follows: Section 2 and Section 3 present the background of antimicrobial drug discovery and sequencing technique development, respectively. Section 4 introduces commonly used DL approaches in the antimicrobial field. Sections 5 and Section 6 discuss current applications of DL in AMR mechanisms prediction and antimicrobial drug discovery, respectively. Section 7 highlights the existing challenges and future perspectives. Finally, Section 8 concludes the paper.

## 2 The evolution of antimicrobial drug discovery

The discovery of antibiotics represents a revolutionary milestone in the history of medicine, fundamentally transforming the management of infectious diseases. In 1928, Scottish scientist Alexander Fleming serendipitously observed that *Penicillium notatum* produced a substance capable of inhibiting the growth of *S. aureus*, thereby heralding the beginning of the antibiotic era (Aminov, 2010; Iskandar et al., 2022). This groundbreaking observation laid the foundation for subsequent advancements, including the successful purification of penicillin by Howard Florey and Ernst Chain in 1940, which enabled large-scale production and clinical deployment (Chain et al., 2005). Prior to the advent of antibiotics, infectious diseases accounted for more than half of all human mortality. The introduction of antimicrobial drugs in the early 20th century led to a dramatic reduction in infection-related deaths and significantly extended life expectancy. Moreover, Antibiotics have served as a cornerstone in the development of modern medicine, providing essential protection against opportunistic and nosocomial infections in interventions such as complex surgical procedures, cancer chemotherapy, and organ transplantation.

After the 1940s, antibiotic discovery entered a “Golden Age” with the systematic screening of soil microorganisms for antimicrobial activity, pioneered by Selman Waksman (Lewis, 2012, 2013; Ribeiro da Cunha et al., 2019). This approach led to the identification of a wide range of clinically valuable antibiotics. During this period, key drugs such as streptomycin (1944) and vancomycin (1958) were successfully discovered and introduced into clinical practice, substantially expanding the therapeutic options for bacterial infections (Levine, 2006; Durand et al., 2019). These antibiotics, primarily derived from the secondary metabolic pathways of microorganisms, exhibit high biological activity, remarkable chemical diversity, and strong target specificity. Through extensive evolutionary selection in natural ecological environments, these compounds have developed into structurally complex molecules with optimized antimicrobial functions. The success of this era firmly established microbial natural products as one of the most productive sources for antibacterial drug development.

Traditional antibiotic discovery has long been based on the “cultivation-screening” model, which involves isolating cultivable microorganisms and evaluating the antimicrobial activity of their metabolic products to identify novel compounds. Although this approach contributed significantly to the development of clinically important antibiotics during the mid-twentieth century, its inherent limitations have become increasingly prominent. The traditional antibiotic discovery pipeline is lengthy and costly. Frequent rescreening of common microbial strains often results in the rediscovery of known compounds. This limits the identification of novel antimicrobial drugs and reduces overall screening efficiency (Baranova et al., 2023). Furthermore, it is estimated that more than 99% of microbial species in natural environments can not be cultured under standard laboratory conditions, resulting in the neglect of vast reservoirs of potentially valuable biosynthetic gene clusters (Lewis, 2013; Wang et al., 2024). Antimicrobial compounds have been isolated from extreme environments, including deep-sea sediments and polar regions. Their development, however, is often constrained by low natural abundance, structural complexity, and challenges in scaling up for industrial production. These factors limit their potential as viable antibiotic candidates. For instance, marinomycin, a macrodiolide polyketide antibiotic belonging to a new structural class of antitumor antibiotics, exhibits potent antibacterial activity but faces significant obstacles in clinical translation due to its synthetic complexity, scalability issues, and an uncertain commercial market (Kwon et al., 2006).

Currently, the global threat of antibiotic resistance has escalated significantly, as pathogenic bacteria continue to develop enhanced adaptability to existing antimicrobial drugs. The emergence and proliferation of multidrug-resistant and even pan-drug-resistant strains have raised concerns regarding the advent of a potential “post-antibiotic era” (Kwon and Powderly, 2021). Superbugs have evolved extensive antibiotic resistance, whereas the development of novel antimicrobials remains insufficient to counteract this rapid adaptation. As of December 2020, only 43 antibiotic candidates were in Phase I to Phase III clinical trials or awaiting regulatory approval (The Pew Charitable Trusts, 2021). This was significantly fewer than the number of anticancer drugs in development during the same period (Cook and Wright, 2022; Ong'uti et al., 2022). Moreover, the need to restrict antibiotic use to prevent resistance development results in low market returns compared with therapies for chronic or oncological diseases, thereby discouraging pharmaceutical investment in antibiotic research and development.

The combination of β-lactam antibiotics with β-lactamase inhibitors has represented an essential strategy for the treatment of infections caused by resistant bacteria since the 1980s (Cook and Wright, 2022). However, the effectiveness of this approach has been compromised over time as β-lactamases have developed resistance to existing inhibitors. While ceftazidime in combination with diazabicyclooctane-based β-lactamase inhibitors such as avibactam demonstrates activity against a range of resistant pathogens, this regimen is not effective against strains expressing metallo-β-lactamases such as NDM-1 (Davies and Everett, 2021; Nordmann et al., 2021). Moreover, the development pipeline for antibiotics targeting Gram-negative pathogens remains severely limited. In particular, treatment options for infections caused by carbapenem-resistant *Enterobacteriaceae* and other multidrug-resistant Gram-negative organisms are extremely scarce. Despite urgent clinical demand, the relatively small patient population fails to support a sustainable commercial model, thereby further impeding the advancement of antibiotic innovation.

With the continuous escalation of AMR, non-antibiotic antimicrobial strategies have attracted increasing attention as potential alternatives or adjuncts to conventional therapies. Antimicrobial peptides (AMPs), typically composed of 10 to 50 amino acids, exhibit broad-spectrum antimicrobial activity and a low tendency to induce resistance. To date, approximately 20,000 natural and synthetic AMPs have been identified. Among them, traditional AMP-based drugs such as bacitracin and polymyxins have received approval from the United States Food and Drug Administration (Kang et al., 2019; Ho et al., 2025). In recent years, several other AMPs, including LL-37, Omiganan, Dalbavancin, Orbactiv, and Pexiganan, have advanced into clinical trials (Ho et al., 2025). Current research increasingly focuses on improving the efficacy, safety, and stability of AMPs through the application of peptidomimetic technique, AI-assisted peptide design, and advanced drug delivery systems. Phage therapy has also garnered renewed interest due to its high specificity, which allows for targeted bacterial elimination without disrupting the host microbiota (El Haddad et al., 2019). Bacteriophages possess the ability to self-replicate in the presence of target bacteria and exhibit self-limiting behavior once the host bacteria are eliminated. Additionally, certain phages encode polysaccharide-degrading enzymes capable of disrupting bacterial biofilms.

In response to the escalating global challenge of antibiotic resistance, conventional antimicrobial drug discovery paradigms have become increasingly inadequate to meet urgent clinical demands. Emerging techniques such as genome sequencing and DL are now revitalizing the development of novel antimicrobial drugs. The integration of these techniques not only enhances the mechanistic understanding of resistance but also offers practical and scalable strategies for the development of novel antimicrobials characterized by high efficacy, low toxicity, and a reduced risk of resistance. Their applications include the *de novo* design of AMPs, virtual screening of compound libraries, prediction of antimicrobial activity, and optimization of lead compounds. In summary, these innovations are reshaping the future landscape of antibiotic discovery and development. A detailed explanation of sequencing and DL techniques are provided in following sections.

## 3 The development of sequencing technique

The development of sequencing technique has undergone several transformative stages, significantly advancing the ability to decode genetic information. In 1953, the double-helix structure of DNA was elucidated by Watson and Crick, laying the theoretical foundation for subsequent sequencing technique (Watson and Crick, 1953). The first generation of DNA sequencing methods emerged in 1977, with the introduction of the chemical degradation method by Walter Gilbert and the chain termination method using dideoxynucleotides by Maxam and Gilbert (1977) and Sanger et al. (1977). These approaches marked the beginning of first-generation sequencing. A major milestone in automation was achieved in 1986 with the introduction of the first automated DNA sequencer, the ABI 370A (also known as the AB 370), by Applied Biosystems, which facilitated more efficient and scalable DNA sequencing (Smith et al., 1986). In 1995, the complete genome of *Haemophilus influenzae* Rd. was sequenced for the first time, highlighting the increasing capacity of genomic technique (Fleischmann et al., 1995). The second generation of sequencing, also known as high-throughput sequencing or next-generation sequencing, represented a revolutionary shift from the traditional Sanger method. It enabled the simultaneous sequencing of hundreds of thousands to millions of DNA fragments. However, the read lengths were substantially shorter, typically ranging from several dozen to several hundred base pairs, compared to nearly 1,000 base pairs with first-generation sequencing methods. This advancement significantly increased both sequencing speed and throughput. The three major second-generation platforms include the 454 pyro-sequencing technique developed by Roche in 2005, the Solexa technique commercialized by Illumina in 2006, and the SOLiD platform introduced by Applied Biosystems in 2007 (Margulies et al., 2005; Shendure et al., 2005; Bentley et al., 2008). The third generation of sequencing technique offers further innovation by enabling direct sequencing of single DNA molecules without the need for PCR amplification. This generation is characterized by long read lengths and real-time sequencing capabilities. Prominent third-generation platforms include single-molecule real-time sequencing developed by Pacific Biosciences (PacBio) and nanopore sequencing technique represented by Oxford Nanopore Technique (Branton et al., 2008; Eid et al., 2009). Sequencing techniques have evolved from low-throughput to high-throughput platforms, and from indirect detection to real-time, direct reading, thereby opening new avenues for their in-depth application in AMR surveillance and mechanistic research.

With the widespread use of antibiotics, the emergence and dissemination of resistant bacterial strains have become a critical global public health challenge. Traditional antimicrobial susceptibility testing (AST), particularly culture-based phenotypic methods, remains the cornerstone of clinical diagnostics. However, these conventional approaches are increasingly limited by prolonged turnaround times, suboptimal sensitivity, and a lack of comprehensive data for timely epidemiological surveillance. Furthermore, inconsistencies across various phenotypic methods and poor reproducibility between laboratories hinder the standardization of AST procedures, thereby constraining the implementation of precision medicine and the effectiveness of public health interventions. The rapid development of sequencing techniques enables in-depth elucidation of AMR mechanisms, facilitates the discovery of novel resistance genes and targets, and supports the identification of resistance gene clusters with high resolution (Wheeler et al., 2023). These molecular insights significantly enhance the ability to detect, monitor, and control resistant pathogens in both clinical and environmental settings.

WGS provides comprehensive genetic information and offers significant advantages in elucidating AMR mechanisms, tracing the transmission routes of resistant strains, and monitoring the horizontal transfer of resistance genes (Walker et al., 2013; Lindsay, 2014; Walker et al., 2015). This technique not only accelerates pathogen identification and diagnostics but also enables a forward-looking understanding of emerging resistant organisms and their evolutionary dynamics, supporting the development of novel antibiotics and the formulation of precision control strategies. Building upon WGS, genome-wide association studies (GWAS) have emerged as a powerful genomic approach for identifying statistical associations between genetic variation and AMR phenotypes. Conventional GWAS typically rely on univariate analyses that assess the effect of individual loci on resistance traits. However, AMR phenotypes are often determined by complex epistatic interactions among multiple genetic variants and influenced by high-frequency recombination and horizontal gene transfer in microbial populations. To address these challenges, microbial GWAS is evolving toward multi-locus modeling and the integration of DL (Jia et al., 2024; Wang et al., 2025). These advanced approaches allow for the simultaneous consideration of combinatorial genetic effects and enable the automatic extraction of latent features from large-scale datasets, leading to the construction of more accurate predictive models and deeper mechanistic insights into AMR. The convergence of these emerging techniques signifies a transition toward a more intelligent, data-driven era in antimicrobial research.

## 4 Brief introduction of deep learning

DL, an emerging branch of machine learning (ML), aims to learn hierarchical representations from complex and high-dimensional data through multilayer neural networks. By applying successive nonlinear transformations, DL models can automatically extract meaningful features and progressively map raw inputs into increasingly abstract representations. This capability has enabled DL to address a wide range of tasks, including classification, regression, prediction, and object detection (Akuthota and Bhargava, 2025; Waqas et al., 2025; Ahmed et al., 2024; Silva et al., 2025). In essence, DL relies on deep neural networks (DNNs) with multiple hidden layers for representation learning. Compared with conventional shallow neural networks, DNNs are capable of learning more hierarchical and abstract representations from complex data.

In recent years, DL architectures have rapidly evolved to accommodate diverse data modalities and learning objectives. Early DL methods mainly focused on discriminative and predictive tasks, aiming to learn feature representations for classification or regression. Subsequently, the development of generative models enabled DL systems to explore complex data distributions and generate novel samples. More recently, large-scale pretrained models, including language models and biological foundation models, have further expanded the capability of DL by learning transferable representations from massive datasets. Accordingly, DL methods can be broadly categorized into three major groups: (i) conventional discriminative and predictive models, including convolutional neural networks (CNNs), graph neural networks (GNNs), ecurrent neural networks (RNNs), multiple long short-term memory (LSTM) networks, and Transformers; (ii) generative models, including variational autoencoders (VAEs), generative adversarial networks (GANs), autoregressive generative models, and diffusion models; and (iii) language models and foundation models, including general-purpose large language models (LLMs), protein language models (PLMs), genomic language models (gLMs), and biological foundation models. The relationship among different biological data modalities, DL model families, and representative applications is summarized in Figure 1.

**Figure 1:**
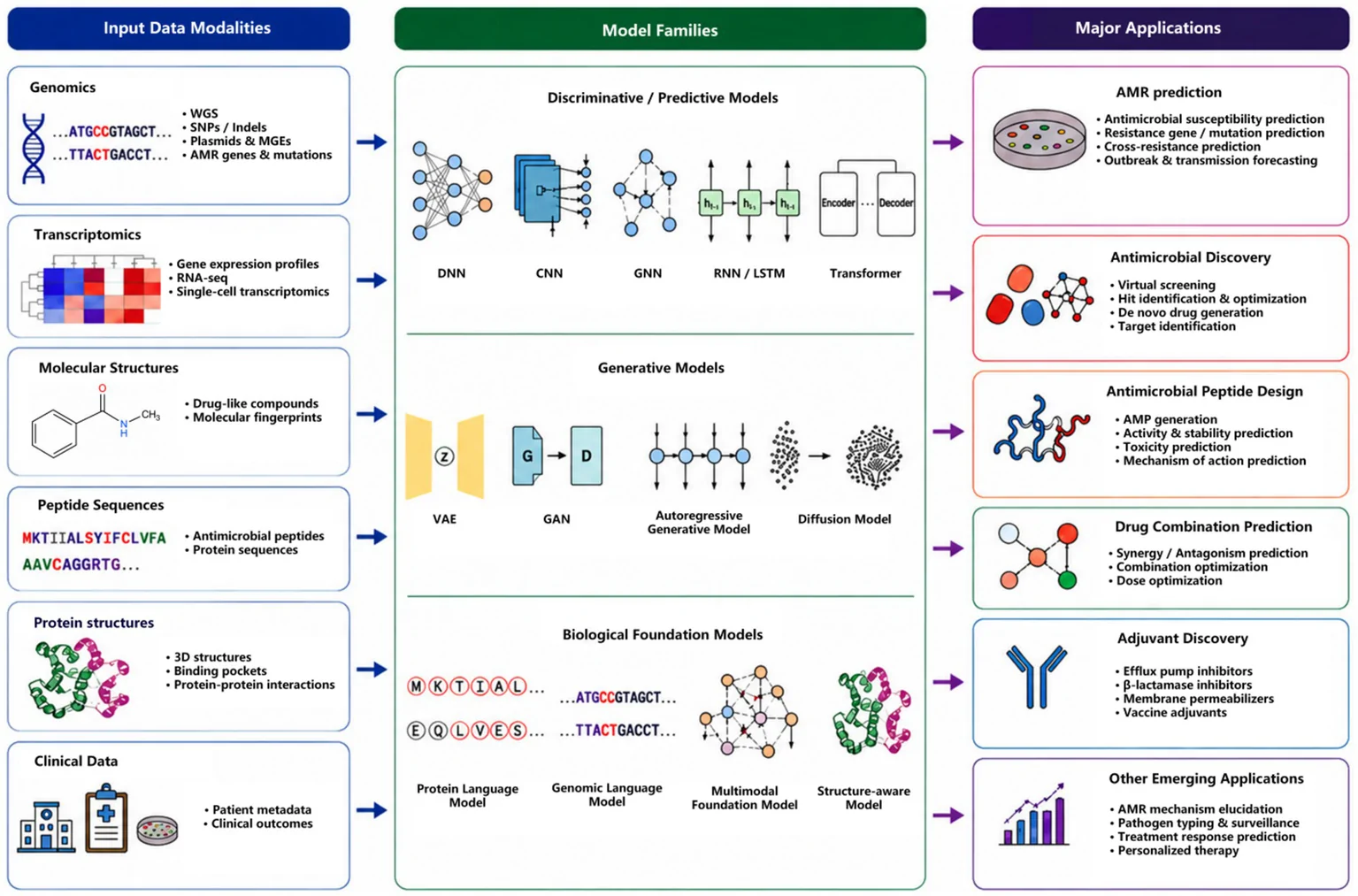
Deep learning for combating antimicrobial resistance.

### 4.1 Discriminative and predictive models

Conventional DL models mainly aim to learn discriminative representations from input data and perform prediction or classification tasks. Among these architectures, CNNs, GNNs, RNNs, LSTM networks, and Transformers represent some of the most influential DL frameworks (Lluka and Stokes, 2023; Zhou et al., 2025a; Cesaro et al., 2023; Yang et al., 2025).

CNNs are among the most representative architectures within DNNs and have achieved remarkable success in processing structured data, particularly images and spatially organized signals (Zheng et al., 2024). The fundamental principle of CNNs is to employ convolutional operations to extract local patterns and hierarchical features from input data. Through stacked convolutional layers, CNNs can progressively capture low-level and high-level representations, while pooling or other downsampling operations can reduce computational complexity and improve feature robustness (LeCun et al., 1998; LeCun et al., 2015). Representative CNN architectures, including LeNet-5, AlexNet, VGG-Net, GoogLeNet, and ResNet, have substantially advanced the field of computer vision and have also been widely adopted in biological data analysis.

GNNs are neural network architectures specifically designed for graph-structured data, where nodes represent entities and edges describe relationships among them. The core mechanism of GNNs is message passing, in which each node iteratively aggregates information from neighboring nodes to update its representation (Scarselli et al., 2009). Through this process, GNNs can effectively capture relational dependencies and structural information within non-Euclidean data, making them suitable for modeling complex biological systems and molecular structures (Zhou et al., 2020).

RNNs are designed for sequential data processing by introducing recurrent connections that allow information from previous time steps to influence current representations. This mechanism enables RNNs to capture sequential dependencies and has led to their applications in time-series analysis and biological sequence modeling (Waqas and Humphries, 2024). LSTM networks, as an improved variant of RNNs, introduce gating mechanisms to regulate information flow and effectively capture long-term dependencies while alleviating the vanishing-gradient problem (Al-Selwi et al., 2024). Due to the sequential characteristics of biological data, RNN- and LSTM-based approaches have been explored for analyzing DNA, RNA, protein sequences, and other ordered biological information.

Transformers represent another important milestone in DL development. Unlike recurrent architectures, Transformers rely primarily on self-attention mechanisms to capture relationships among different elements within a sequence and effectively model long-range dependencies (Vaswani et al., 2017). The incorporation of positional encoding, feed-forward networks, residual connections, and layer normalization enables efficient feature extraction and stable optimization. Compared with traditional recurrent models, Transformers support highly parallelized computation and have become a dominant architecture for large-scale sequence modeling.

### 4.2 Generative deep learning models

While conventional DL models mainly focus on discriminative or predictive tasks, generative deep learning models aim to learn the underlying probability distribution of training data and generate new samples. This capability provides an important advantage when the objective is not only to analyze existing data but also to explore previously unknown biological or chemical spaces. Major generative DL frameworks include VAEs, GANs, autoregressive generative models, and diffusion models.

VAEs are probabilistic generative models based on an encoder–decoder architecture. The encoder maps input data into a continuous latent representation, while the decoder reconstructs original samples or generates new samples from the learned latent distribution (Kingma and Welling, 2014). By learning meaningful latent spaces, VAEs enable efficient exploration of complex biological sequence and molecular spaces.

GANs represent another important category of generative models and consist of two competing components: a generator and a discriminator. During training, the generator attempts to produce realistic samples, whereas the discriminator distinguishes generated samples from real data. Through adversarial optimization, GANs can capture complex data distributions and generate high-quality synthetic samples (Goodfellow et al., 2014).

Autoregressive generative models formulate data generation as a sequential prediction process, where each element is generated based on previously generated elements (Vaswani et al., 2017). This strategy is particularly suitable for biological sequences because DNA, RNA, and protein sequences contain inherent sequential dependencies. By modeling conditional probabilities among sequential elements, autoregressive approaches can generate novel biological sequences.

Diffusion models have recently emerged as powerful generative frameworks by learning the reverse denoising process from progressively corrupted data. Compared with traditional generative approaches, diffusion models can capture complex data distributions and have shown promising potential for generating molecules, protein structures, and biological sequences (Ho et al., 2020).

Collectively, generative DL models extend the role of artificial intelligence from prediction toward creation by enabling the exploration and generation of new biological entities. These approaches complement conventional discriminative models and provide new computational strategies for biological design.

### 4.3 Biological language models and foundation models

Transformers have also initiated a new era of language modeling. The self-attention mechanism and large-scale pretraining capability allow Transformer-based models to learn generalizable representations from massive unlabeled datasets.

The emergence of Transformer-based architectures marks the beginning of the era of LLMs. Based on this architecture, researchers have developed language models with billions or even trillions of parameters, including GPT (Brown et al., 2020; Koubaa, 2023), PaLM (Chowdhery et al., 2023), and LLaMA (Touvron et al., 2023). These models have demonstrated remarkable performance in natural language understanding, text generation, reasoning, and multimodal information processing. The success of general-purpose LLMs highlights the ability of Transformer architectures to learn transferable representations and provides a foundation for developing domain-specific foundation models.

Inspired by the success of NLP-based LLMs, researchers have extended Transformer-based language modeling approaches to biological sequences, including DNA, RNA, and proteins. Biological sequences can be regarded as a biological language, where nucleotides or amino acids act as basic tokens and their sequential arrangements encode information associated with biological structure and function.

Protein language models (PLMs) apply self-supervised learning strategies to large-scale protein sequence datasets and learn contextual representations without requiring extensive manual annotation. Representative PLMs, including ProtBERT, ProtT5, and the Evolutionary Scale Modeling (ESM) family, have demonstrated the ability to capture evolutionary, structural, and functional information encoded within protein sequences (Elnaggar et al., 2022; Lin et al., 2023). These learned representations can subsequently support various downstream biological tasks.

Similarly, genomic language models (gLMs) employ Transformer architectures to model DNA sequences by treating nucleotide sequences as biological tokens. DNABERT demonstrated that pretrained genomic representations could capture meaningful sequence patterns and improve downstream genomic analysis tasks (Ji et al., 2021). More recent genomic models have further expanded this concept by incorporating larger genomic contexts and more complex biological information.

The development of biological language models has further contributed to the emergence of biological foundation models. Unlike conventional DL models optimized for specific tasks, foundation models are pretrained on large-scale heterogeneous datasets and can be adapted to multiple downstream tasks through fine-tuning, transfer learning, or other adaptation strategies. By integrating diverse biological modalities, including genomic sequences, transcriptomic profiles, protein sequences, molecular structures, and clinical data, biological foundation models provide a general framework for learning transferable biological representations.

Beyond sequence-based biological language models, structure-aware models have recently emerged by incorporating three-dimensional molecular information. Protein structures provide critical insights into molecular mechanisms and therapeutic interactions that cannot be fully inferred from sequence information alone. AlphaFold2 demonstrated the capability of deep learning models to predict protein structures with high accuracy (Jumper et al., 2021), while ESMFold further integrated protein language modeling with structure prediction to enable rapid structural analysis at large scale (Lin et al., 2023). These structure-aware approaches provide new opportunities for antimicrobial target identification, resistance mechanism analysis, and structure-based antimicrobial drug discovery. In antimicrobial research, biological foundation models provide a promising computational framework beyond conventional predictive approaches. By leveraging large-scale biological knowledge, these models can facilitate antimicrobial resistance prediction, resistance gene identification, antimicrobial peptide design, molecular optimization, and discovery of novel antimicrobial candidates.

Overall, DL has evolved from task-specific predictive models toward large-scale generative and foundation-model-based paradigms. The integration of generative models, biological language models, and foundation models represents a major advancement in computational biology and provides a powerful basis for future data-driven antimicrobial research.

## 5 Application of deep learning in predicting antimicrobial resistance mechanisms

DL techniques have garnered significant attention for their ability to predict AMR mechanisms across various bacterial species, particularly in MTB. In recent years, with the vigorous development of DL, an increasing number of approaches have been applied to the prediction of AMR. DNNs, through their densely connected neurons across multiple hidden layers, are capable of learning complex features from genomic data that are associated with AMR. To address the limitations of the “best-hit” approach, which rely on sequence searches against existing databases for identifying or predicting antibiotic resistance genes (ARGs) and typically result in high false-negative rates. Arango-Argoty et al. (2018) constructed two DL models, DeepARG-SS and DeepARG-LS, specifically designed for short read sequences and full gene length sequences, respectively. The evaluation of these models across 30 antibiotic resistance categories demonstrated that the DeepARG models achieved high precision (> 0.97) and recall (> 0.90) in predicting ARGs. Compared to the typical hit approach, the DeepARG models showed superior performance, yielding consistently lower false-negative rates and thereby improving the overall recall (> 0.90). Resistance co-occurrence with first-line anti-tuberculosis drugs is a common phenomenon. However, current analysis methods based on MTB genetic data have limitations. They are unable to account for the resistance co-occurrence and cannot capture the underlying structure of genomic data corresponding to different lineages. In view of this, Yang et al. (2019) proposed an end-to-end multi-task model based on a deep denoising autoencoder (DeepAMR) for multi-drug classification. The results indicated that DeepAMR outperformed both the baseline model and four ML models. It achieved an average area under the receiver-operating characteristic (AUROC) of over 94.4% in predicting resistance to the four first-line anti-tuberculosis drugs. The diagnosis of multidrug-resistant and extensively drug-resistant tuberculosis is a priority in global health. Chen et al. (2019) presented the MD-WDNN model to determine the resistance of MTB isolates to 10 anti-tuberculosis drugs based on whole-genome sequencing data. This model was trained on rare and common genetic variations and achieved the best performance on a large and comprehensive tuberculosis dataset, with a prediction time of less than one-tenth of a second. This demonstrated the effectiveness of the proposed model as a tool for diagnosing drug resistance in MTB. The authors claimed that MD-WDNN was the first multi-drug model to incorporate a large number of genotypic predictors, which were highly important for determining resistance to one or more drugs (Chen et al., 2019).

As one of the most well-known and widely applied models in DL community, CNN has also demonstrated excellent performance in AMR prediction. Unlike FC neural networks, each neuron in a CNN is connected only to a portion of the input data, known as the receptive field. This local connectivity helps reduce the number of parameters and enables the model to extract key features from the data more efficiently. This capability is particularly useful for handling high-dimensional and complex high-throughput genetic data. Currently, there have been many studies reported on the use of CNNs for AMR prediction. Li Y. et al. (2021) proposed a DL-based hierarchical multi-task method called HMD-ARG (Figure 2), in which each layer of the prediction model is a CNN. This allowed for detailed annotation of ARGs from three important aspects: antibiotic resistance categories, resistance mechanisms, and gene mobility. The authors firmly believed that HMD-ARG could serve as a powerful tool for identifying ARGs and thus help mitigate their global threat. As indicated in recent studies, most existing computational approaches for identifying AMR genes (ARGs) are primarily based on sequence alignment and similarity to known ARG sequences. Such approaches are limited by the choice of reference database and may miss novel ARGs. For this reason, a recent study developed ARGNet (Pei et al., 2024), which combined an unsupervised learning autoencoder model for identifying ARGs with a multi-class classification CNN that categorized ARGs without relying on sequence alignment. The results demonstrated that ARGNet outperformed other DL models, including DeepARG and HMD-ARG, in most application scenarios, particularly in quasi-negative testing and in prediction consistency analysis based on the phylogenetic tree. In addition, ARGNet had a shorter inference runtime compared to other methods. Some studies have found that most current methods focus only on predicting antibiotic categories, while neglecting other important properties of ARGs. Moreover, existing methods fail to consider the causal relationships between these attributes, thereby limiting predictive performance. Zhao et al. (2024) proposed a causality-oriented ARG attribute annotation framework called CausalARG, which was a multi-layer CNN that used a Gaussian mixture model to describe hidden biological patterns determining the properties of ARGs and applied a causal representation learning process to derive latent features. Mass spectroscopy has been widely used in routine clinical microbiology laboratories for identifying bacterial species and detecting AMR. DL-based analysis of AMR is still emerging, and most models rely on spectral filtering and preprocessing techniques. Lopez-Cortes et al. (2024) proposed a CNN model, MSDeepAMR, that learns directly from raw mass spectra to predict AMR. The MSDeepAMR model was applied to *Escherichia coli*, *Klebsiella pneumoniae*, and *S. aureus.* The results revealed that the model achieved strong performance, with AUROC above 0.83 in most case studies.

**Figure 2:**
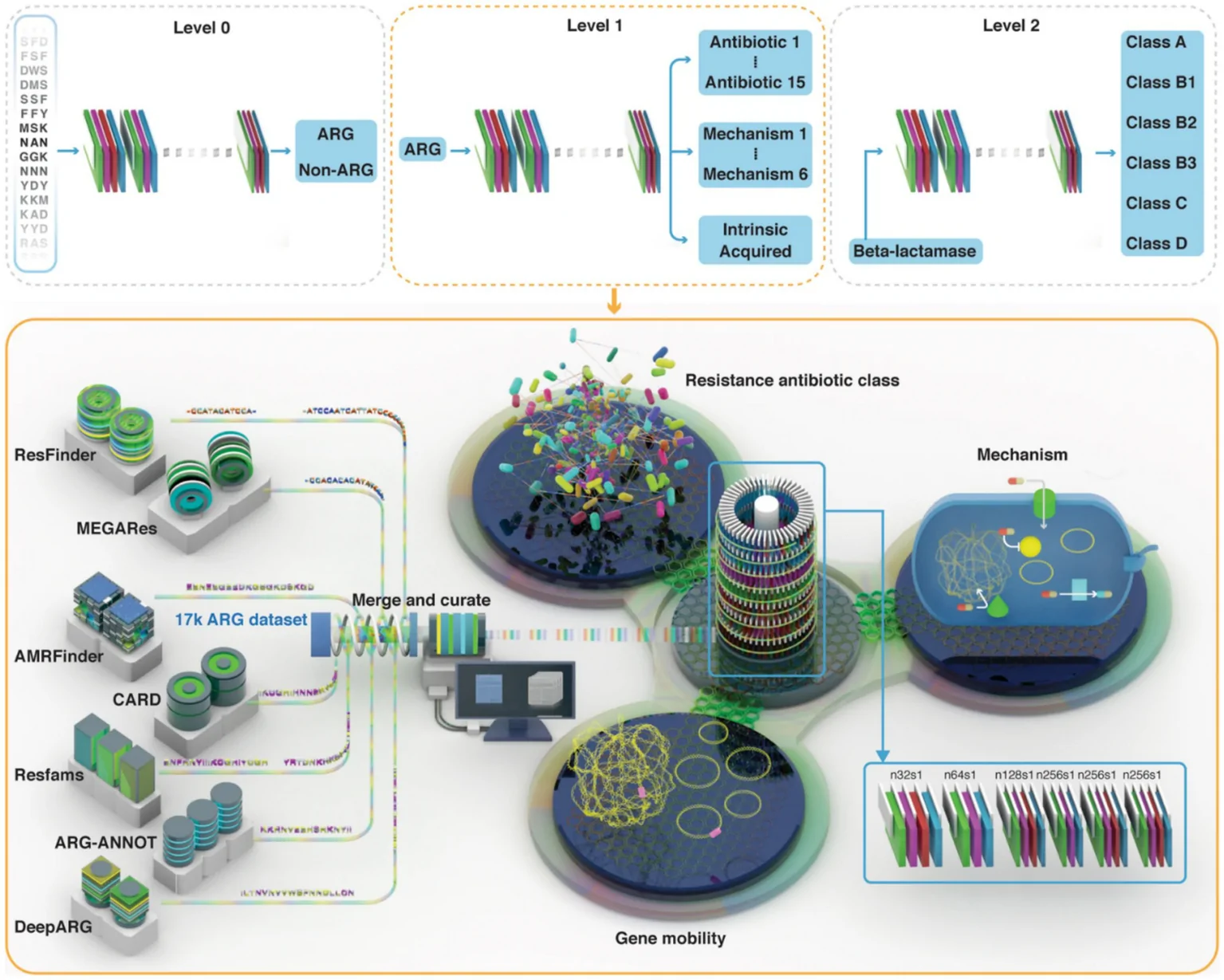
The HMD-ARG proposed by Li Y. et al. (2021) consists of three DL models for a three-level prediction process, and each level of the model is constructed based on convolutional neural network. The Level 0 model determines whether the input sequence is an ARG. If it is, a multi-task model performs Level 1 prediction, identifying the antibiotic resistance family, resistant mechanism, and gene mobility information simultaneously. If the result from Level 1 is predicted as beta-lactamase, the sequence is then input into the Level 2 model to predict its specific beta-lactamase subclass. Reproduced from Li Y. et al. (2021) under the terms of the Creative Commons Attribution 4.0 International License (CC BY 4.0), https://creativecommons.org/licenses/by/4.0.

RNNs and LSTMs have shown excellent performance in various sequence data processing tasks, such as NLP, financial data analysis, and weather forecasting. In recent years, they have also demonstrated strong capabilities in handling omics data and predicting AMR. Hamid and Friedberg (2019) developed an RNN model to predict bacteriocins, which are antimicrobial peptide products produced by bacteria and are considered candidates for expanding the available options for antimicrobial drugs. Bacteriocins are difficult to discover using sequence similarity methods because they are often non-homologous. Their approach did not rely on sequence similarity searches and was successful in identifying several putative bacteriocins with high probability. Another study (Kromer-Edwards et al., 2022) designed an RNN model to predict minimum inhibitory concentration (MIC) values, which determine whether bacteria are classified as susceptible or resistant to antibiotics based on breakpoints. DL-based methods for predicting MIC values offer a promising solution to overcome the inefficiencies of traditional methods, which are typically labor-intensive and time-consuming. The experimental results showed that, compared to other ML models, the RNN model achieved an average F1 score of 0.75, whereas the other ML models attained average F1 scores of 0.67 and 0.58, respectively. Some researchers believed that identifying new plasmids could help guide the discovery of novel antibiotic resistance genes, toxins, and genes directly involved in host–microbe interactions. Andreopoulos et al. (2022) developed DeepPlasmid, an LSTM-based model designed to identify plasmids from bacterial chromosomes based on DNA sequences and their encoded biological features. It required only assembled sequences generated by any sequencing platform and assembly algorithm as input, with a runtime that scaled linearly with the number of assembled sequences. DeepPlasmid achieved an AUC-ROC of over 89%, making it more accurate than five other plasmid classification methods.

In recent years, a more advanced neural network, Transformer, has emerged in the DL community. Initially developed for NLP, it has since demonstrated remarkable performance across many fields. Similarly, in the field of AMR prediction, several studies have reported the application of Transformer models. Wu et al. (2023) proposed a Transformer-based framework (i.e., PLM-ARG) for ARG identification that utilized a pre-trained large protein language model to simultaneously perform ARGs detection and resistance class classification. In addition, the authors validated the PLM-ARG model using an independent validation set, achieving a Matthew’s correlation coefficient of 0.838, which outperformed other publicly available ARG prediction tools by improvements ranging from 51.8 to 107.9%. Dong et al. (2024) proposed a Transformer-based model, TGC-ARG, for ARGs prediction. The framework took protein sequences as input and employed feature extraction techniques to extract distinct features from both sequence and structural data. It performed ARGs prediction through contrastive learning and an ensemble MLP. Experimental results indicated that their method outperformed the existing SOTA methods. The representative studies are summarized in Table 1.

**Table 1:** DL algorithms for predicting antimicrobial resistance mechanisms.

Purpose	ARG or plasmid database	Type of input data	Proposed method	References
Predicting antibiotic resistance genes from metagenomic data	Merging database of CARD, ARDB, and UNIPROT (14,933 sequences)	Short read sequences and full gene length sequences	DeepARG-SS and DeepARG-LS (DNN)	Arango-Argoty et al. (2018)
Predicting co-occurrent resistance of *Mycobacterium tuberculosis*	WGS and DST phenotypes from 8,388 isolates	Genomic mutations	DeepAMR (DNN)	Yang et al. (2019)
Predicting *Mycobacterium tuberculosis* resistance	ReSeqTB knowledgebase (1,379 MTB isolates)	Short read sequences	MD-WDNN (DNN)	Chen et al. (2019)
ARGs annotations from three important aspects: resistant antibiotic class, resistant mechanism, and gene mobility.	HMD-ARG-DB database including CARD, AMRFinder, ResFinder, ARG-ANNOT, DeepARG, MEGARes, and Resfams (17,282 sequences)	Protein sequence	HMD-ARG (CNN)	Li Y. et al. (2021)
Robust identification and classification of ARGs	ARGNet-DB database including CARD, AMRFinder, ResFinder, Megares, DeepARG and HMD-ARG (27,464 sequences)	Protein sequence	ARGNet (CNN)	Pei et al. (2024)
Annotating properties of antibiotic resistance genes	HMD-ARG-DB database (17,282 sequences)	Protein sequence	CausalARG (CNN)	Zhao et al. (2024)
Predicting antimicrobial resistance	DRIAMS database (300,000 mass spectra)	Raw mass spectra	MSDeepAMR(CNN)	Lopez-Cortes et al. (2024)
Identifying antimicrobial peptides	BAGELand Uniprot-Swissprot database (346 sequences)	Protein sequence	RNN	Hamid and Friedberg (2019)
Predicting minimum inhibitory concentration for *K. pneumoniae*	SENTRY Program (5,085 isolates)	Genome sequence	RNN	Kromer-Edwards et al. (2022)
Separating plasmids from bacterial chromosomes	RefSeq.bacteria dataset (40,000 sequences) ACLAME dataset (1,056 sequences) PLSDB database (2,7,939 sequences)	Chromosomal sequence and plasmid sequence	Deeplasmid (LSTM)	Andreopoulos et al. (2022)
ARG identification and resistance category classification	Merging database of CARD, ResFinder, MEGARes, AMRFinderPlus, and HMD-ARG-DB (26,391 sequences)	Protein sequence	PLM-ARG (Transformer)	Wu et al. (2023)
Anticipating antibiotic resistance	ARSS dataset including ResFinder, DeepARG, ARG-annot, Resfams, COALA, MEGARes, and CARD (28,117 sequences)	Protein sequence	TGC-ARG (Transformer)	Dong et al. (2024)

Although DL approaches have substantially improved AMR prediction, different architectures exhibit distinct advantages depending on the characteristics of input data and prediction tasks. Sequence-based models, including DNNs and CNNs, have demonstrated strong capabilities in extracting informative patterns from genomic and protein sequences, enabling efficient ARG identification and resistance prediction. However, their performance is often dependent on the availability and diversity of reference datasets, which may limit the identification of highly divergent or novel resistance determinants. In contrast, Transformer-based models and protein language models can capture long-range dependencies and contextual information from large-scale sequence data, providing new opportunities for identifying previously uncharacterized resistance genes. Nevertheless, the clinical translation of these approaches remains constrained by issues such as dataset heterogeneity, limited prospective validation, and insufficient model interpretability.

## 6 Application of deep learning in antimicrobial drug discovery

### 6.1 Antibiotics

Antibiotics, as key drugs for treating bacterial infections, are facing the severe challenge of increasing drug-resistant bacteria. Despite the worsening antibiotic resistance crisis, only 29 new antibiotics have received marketing authorization since 2010 (BD, 2025), which is insufficient to meet the urgent need for combating multidrug-resistant bacteria. In this context, emerging techniques such as DL provide promising avenues for the development of novel antibiotics. By simulating molecular structures, predicting antibacterial activities and optimizing candidate compounds, DL technology is expected to significantly shorten the drug screening time, improve the discovery efficiency and reduce the research and development cost. For example, virtual screening based on computational models can complete tasks in a few days that would traditionally take months or even years. It accelerates drug discovery, reduces the time and costs associated with traditional methods, and provides a practical solution to the global antibiotic resistance crisis (Table 2).

**Table 2:** DL algorithms for antimicrobial drug discovery.

Type of antimicrobial drug	Database	Type of input data	Proposed method	References
Antibiotic	FDA drug library, natural products library and ZINC15 database	SMILES string	DNN	Stokes et al. (2020)
Antibiotic	High-throughput screening sub-libraries of Broad Institute and the updated drug repurposing hub	SMILES string	GNN	Liu et al. (2023)
Antibiotic	CheMBL database	SMILES string	CNN	Boulaamane et al. (2024)
Antibiotic	FDA drug library and natural products library database	SMILES string	FIAMol-AB (GNN and CNN)	He et al. (2024)
Antibiotic	Mcule purchasable database and Broad Institute database	SMILES string	GNN	Wong et al. (2024)
Antibiotic	FDA drug library, natural products library database, Broad Institute database and the updated drug repurposing hub	SMILES string	MFAGCN (GNN)	Lin et al. (2025)
Antimicrobial peptide	AMP, DRAMP and MilkAMP databases	Peptide sequence	PGAT-ABPp (GNN)	Hao et al. (2024)
Antimicrobial peptide	Thirty-five AMP databases including APD3, dbAMP, DRAMP, etc.	Peptide sequence	iAMPCN (CNN)	Xu et al. (2023)
Antimicrobial peptide	APD3 and DADP databases	Peptide sequence	AMPd-Up (RNN)	Li et al. (2024)
Antimicrobial peptide	CAMP, DBAASP, DRAMP, and YADAMP databases	Peptide sequence	LSTM	Wang et al. (2021)
Antimicrobial peptide	dbAMP, DRAMP, DBAASP, AVPdb, AntiCP and AntiFP database	Peptide sequence	Transformer	Pang et al. (2022)
Antimicrobial peptide	APD and UniProt database	Peptide sequence	ACEP (CNN and LSTM)	Fu et al. (2020)
Bacteriophage	PredPHI, PhageHosts, NCBI, MVP, and PHISDetector databases	Genome sequence	DeepPBI-KG (DNN)	Wei et al. (2024)
Bacteriophage	INPHARED, and NCBI nr databases	Genome sequence	SpikeHunter (DNN)	Yang Y. et al. (2024)
Bacteriophage	NCBI and PHASTER databases	Genome sequence	Seeker (LSTM)	Auslander et al. (2020)
Bacteriophage	PhaLP and NCBI databases	Amino acid sequence	DeepMineLys (CNN)	Fu et al. (2024)
Enzyme inhibitors	ZINC database	SMILES string	RNN-CNN	Shi et al. (2020)
Enzyme inhibitors	PubChem database	Two and three-dimensional physicochemical descriptors	Feed-forward neural network	Bagdad et al. (2024)

“SMILES” is the abbreviation of Simplified Molecular Input Line Entry System, which provides a concise way to describe the structural information of molecules, including atomic types, connection sequences, and types of chemical bonds, etc. SMILES strings can be used by researchers to record and share information about these compounds, conduct database searches, predict chemical reactions, design new drugs, or analyze molecular properties.

In recent years, numerous studies have reported the immense potential of DL in assisting antibiotic discovery. Stokes et al. (2020) trained a DNN capable of predicting molecules with antimicrobial activity. The authors applied this model to screen multiple chemical libraries and identified a molecule (i.e., halicin), whose structure is distinct from that of traditional antibiotics. Halicin demonstrated bactericidal activity against a wide phylogenetic spectrum of pathogens, including MTB and carbapenem-resistant *Enterobacteriaceae*. However, halicin remains a preclinical antimicrobial candidate, and further studies are required to evaluate its pharmacological properties, safety, and clinical potential. This study highlighted how DL could identify structurally novel antimicrobial compounds, thus expanding the chemical space of potential antimicrobial candidates and demonstrating the utility of DL-based approaches in early-stage antibiotic discovery. Liu et al. (2023) screened approximately 7,500 molecules *in vitro* for their ability to inhibit the growth of *Acinetobacter baumannii*. They used this growth inhibition dataset to train a GNN and predicted new structural molecules with activity against *A. baumannii*. The authors claimed to have discovered an antibacterial compound called abaucin, which exhibits narrow-spectrum activity against *A. baumannii*. Abaucin showed antibacterial efficacy in a mouse wound infection model, supporting its potential as a lead compound for further development. Nevertheless, it remains at the preclinical stage and requires additional evaluation before clinical translation. Another study developed a CNN model for discovering antibiotics to treat *A. baumannii* infections (Boulaamane et al., 2024). The results showed that the CNN model performed exceptionally well, outperforming traditional ML models such as random forest and support vector machine. Moreover, this study demonstrated the potential of curcuminoid compounds as antimicrobial candidates against *A. baumannii* infection. To enhance the effectiveness of antibiotic discovery, He et al. (2024) proposed FIAMol-AB, a DL model that integrates GNNs, text convolutional networks, and molecular fingerprinting techniques. The method also incorporates an attention mechanism to fuse multiple forms of information within the model. Experimental results demonstrated that FIAMol-AB achieved competitive performance. By integrating multi-view information and the attention mechanism, FIAMol-AB might better capture complex molecular structures, which potentially improved both the precision and efficiency of antibiotic discovery.

Although DL approaches facilitate the exploration of chemical space, they often suffer from the “black box” problem, lacking transparency and failing to provide meaningful insights into chemical mechanisms. To address this issue, Wong et al. (2024) developed an interpretable, substructure-based method to test this hypothesis, aiming to enable effective, DL-guided exploration of chemical space. The antibiotic activity and human cytotoxicity of 39,312 compounds were experimentally determined, and ensembles of GNNs were applied to predict the antibiotic activity and cytotoxicity of 12,076,365 compounds. The results demonstrated that the method could identify molecular structures with antibiotic potential under the guidance of DL, and also proved that ML models in drug discovery could achieve good interpretability. In the early stages of new drug discovery, identifying structurally unique candidate molecules is crucial for accelerating the research process and reducing costs. A recent study (Lin et al., 2025) proposed a graph convolutional neural network method named MFAGCN. This method integrated three types of molecular fingerprints (including MACCS, PubChem, and ECFP) along with molecular graph representations as input features, aiming to predict the antibacterial efficacy of molecules. The MFAGCN method enabled rapid screening of molecules with effective antibacterial properties and facilitated the identification of functional groups that influenced antimicrobial performance.

Building upon these advancements, LLMs have recently begun to play a role in the field of antimicrobial research, demonstrating unprecedented capabilities in accelerating the discovery, design, and optimization of antibiotics. A recent paper systematically reviewed the critical applications of LLMs in antimicrobial peptide design, highlighted their ability to learn natural peptide sequence patterns, generate novel molecules with potential bioactivity, and predict key properties such as biological activity and toxicity (Guan et al., 2025). These advances significantly improved the efficiency of antibiotic development and offered a promising new strategy for combating continuously evolving pathogens.

Compared with conventional descriptor-based ML approaches, DL methods have expanded antibiotic discovery by enabling direct learning from molecular representations, including SMILES strings and molecular graphs. DNN-based approaches have successfully identified structurally novel antibacterial compounds, whereas GNN-based methods provide advantages in modeling molecular topology and capturing atom-level interactions. More recently, multimodal architectures integrating molecular fingerprints, graph representations, and attention mechanisms have been developed to incorporate complementary chemical information. However, despite promising computational performance, most DL-based antibiotic discovery studies remain limited by insufficient experimental validation, narrow chemical spaces, and uncertainty regarding the biological mechanisms underlying predicted candidates.

### 6.2 Antimicrobial peptides

The overuse of antibiotics has led to the development of antibiotic resistance in nearly all documented bacterial pathogens. This trend results in an increasing demand for new antibiotics, while the arsenal of available antimicrobial drugs is diminishing. AMPs represent a promising class of antimicrobial drugs. They are distinguished by their rapid bactericidal activity, broad-spectrum efficacy, capacity to modulate host immune responses, and reduced likelihood of promoting resistance development. The identification of AMP in wet laboratories requires complex designs, long screening cycles and strict conditions, which makes large-scale screening very difficult (Hancock et al., 2021). With advances in computer science, computational methods now offer the potential for high-throughput preliminary screening prior to clinical testing, providing a novel and efficient avenue for the discovery and identification of AMPs. At present, numerous studies have reported the successful application of DL approaches in AMPs design and identification (Table 2).

Recently, a study proposed a graph neural network (GNN)-based model named PGAT-ABPp (Figure 3). The model utilized structural predictions from AlphaFold2 and features extracted from the pre-trained protein language model ProtT5-XL-U50 (ProtT5) to construct graph representations (Hao et al., 2024). A graph attention network was then employed to learn global discriminative features from the graphs. The results demonstrated that ProtT5 provided significant advantages in AMP identification, and the incorporation of spatial information further enhanced the model’s prediction performance. Xu et al. (2023) constructed a comprehensive AMP dataset and proposed a new DL model, iAMPCN, based on CNN to identify AMP and its related 22 functional activities. Experimental results showed that iAMPCN significantly improved the prediction performance for both AMP identification and corresponding functional activity prediction using four sequence-based features. Results from benchmarking experiments on an independent test set revealed that iAMPCN outperformed many state-of-the-art (SOTA) methods in predicting both the existence of AMPs and their functional activities.

**Figure 3:**
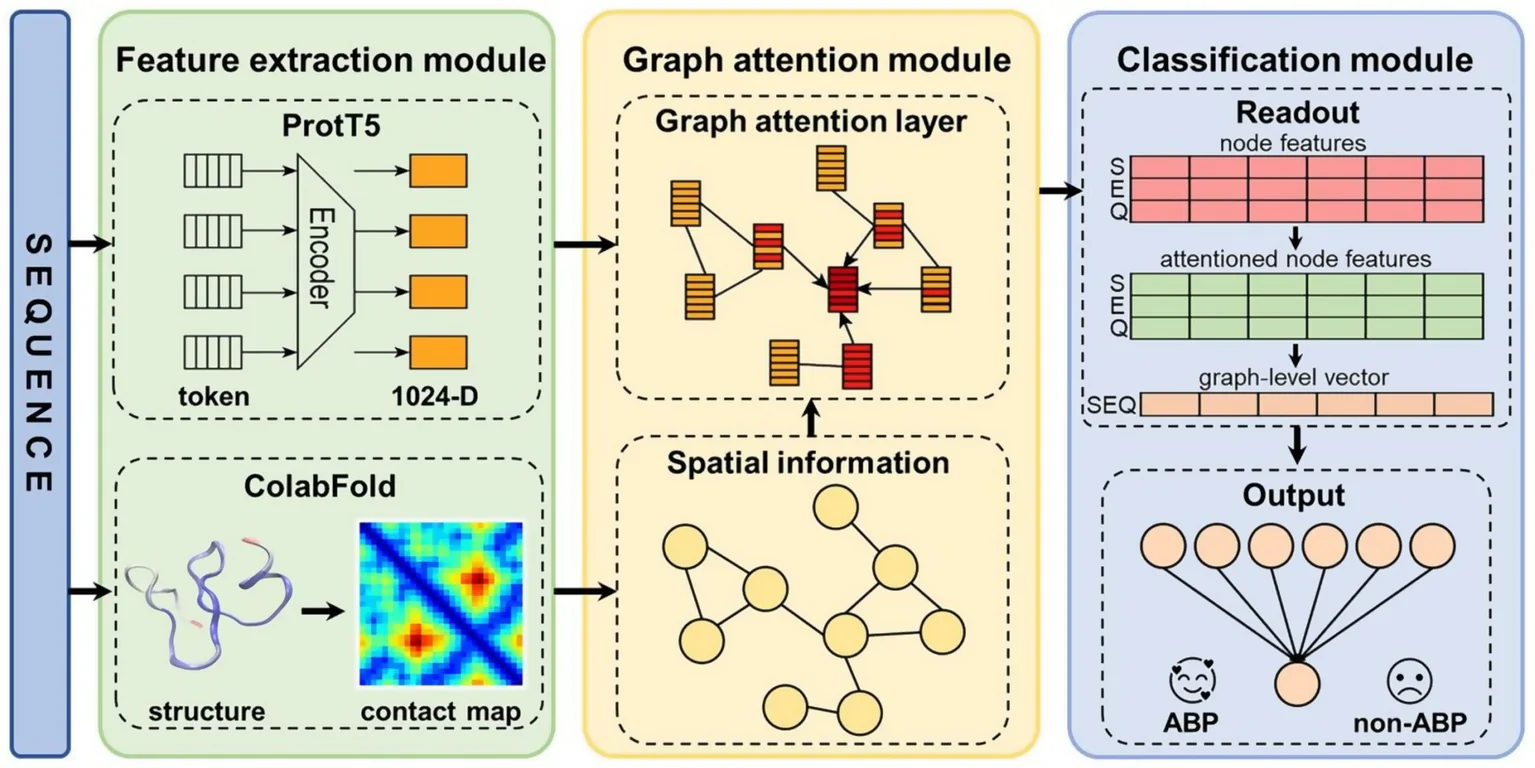
The framework of PGAT-ABPp for antibacterial peptide (ABP) also known as antimicrobial peptide (AMP) identification (Hao et al., 2024). First, ColabFold is used to predict the 3D structure of the protein and generate a contact map. ProtT5 extracts node embeddings, which are combined with spatial information to construct a graph. The graph attention layer updates the node representations, while the readout layer extracts a graph-level vector using an attention mechanism and pooling techniques. Finally, the output layer performs sequence prediction based on this vector. Reproduced from Hao et al. (2024) under the terms of the Creative Commons Attribution 4.0 International License (CC BY 4.0), https://creativecommons.org/licenses/by/4.0.

More recently, the development of AMP-related data resources has provided additional support for computational AMP research. Zhou et al. (2025b) developed the Antibacterial Peptides DataBase (ABPDB) by integrating existing antibacterial peptide databases. ABPDB contains 4,872 antibacterial peptides together with sequence information, activity information, physicochemical properties, and structural information. Three-dimensional structures were also predicted using AlphaFold2. This resource provides a more comprehensive data foundation for computational antibacterial peptide research.

In addition, Zhou et al. (2025a) systematically reviewed available AMP data resources and DL models for AMP prediction, and discussed the limitations and challenges of existing computational approaches. This work highlights the importance of both reliable data resources and appropriate DL models for improving computational AMP prediction (Zhou et al., 2025a).

Sequence models have also proven highly successful in the design and identification of AMPs. This is because the formation of natural proteins or peptides can be analogized to natural language, enabling sequence models from the DL community to directly and accurately decipher information within peptide sequences. Li et al. (2024) proposed AMPd-Up, an RNN-based tool for *de novo* AMP design, demonstrating its practical advantages over existing methods. Candidate sequences generated by AMPd-UP were experimentally validated through antimicrobial susceptibility testing, with 40 out of 58 candidates showing antimicrobial activity against *Escherichia coli* and/or *S. aureus*. These results indicated that AMPd-Up could be effectively used to design novel synthetic AMPs with potent activity. Another study proposed the design of short AMP sequences with potential antibacterial activity against *Escherichia coli* by constructing a LSTM-based generative model and a bidirectional LSTM classification model (Wang et al., 2021). The predicted three-dimensional conformation of the selected short AMPs shows an α-helical structure with amphiphilic surfaces, indicating that LSTMs are effective tools for generating novel AMPs against the target bacteria. Pang et al. (2022) presented a approach based on the Transformer neural network architecture and NLP techniques to extract information from peptide sequences. The authors applied this approach to two AMP-related tasks: the first task involved distinguishing AMPs from other peptides, while the second aimed at identifying the functional activities of AMPs across seven different targets, i.e., gram-negative bacteria, gram-positive bacteria, fungi, viruses, cancer cells, parasites, and mammalian cell inhibitors. The results indicated that this approach achieved the best performance (with a balanced accuracy of 96.85%) in the first task and exhibited a more unbiased prediction for the second task (with an average balanced accuracy of 79.83% across all functional activities). These findings highlight the importance of not only accurately identifying AMPs but also predicting their diverse functional profiles. Effective feature extraction is crucial for improving AMP identification, as it enables models to capture meaningful patterns from peptide sequences that distinguish AMPs from non-AMPs. A previous study designed a novel neural network architecture by combining convolutional layers and LSTM layers (Fu et al., 2020). Leveraging the attention mechanism from NLP, the model identifies important motifs within sequences and employs a “convolution and concatenation” (CVCA) layer to fuse features. The results indicated that this model could effectively capture sequence characteristics and offered significant advantages in the identification of AMPs.

Beyond conventional sequence-based models, recent work has begun to integrate protein language models, structural information, and contrastive learning for AMP prediction. Zhou et al. (2026) proposed CLABP, a contrastive learning-based framework for antibacterial peptide identification. The model integrated ProtT5 embeddings and structural information, including backbone dihedral angles and predicted secondary structures as node features, together with inter-residue distance, motion vectors, and rotation quaternions as edge features. Node and edge representations were independently encoded and aligned into a shared latent space through contrastive learning, followed by feature fusion using a dual cross-attention mechanism. CLABP achieved an accuracy of 92.6% and a Matthews correlation coefficient of 0.853, while ablation analyses supported the importance of edge features and the alignment mechanism (Zhou et al., 2026). More broadly, recent reviews have expanded the scope of AI-driven AMP research beyond prediction alone. Lv et al. (2026) summarized recent AI applications in AMP discovery from four perspectives: AMP prediction, AMP generation, AMP mining, and AMP optimization. The review further discussed the remaining challenges and future directions of AI-driven AMP discovery, indicating that computational AMP research is increasingly extending from identification toward generation, mining, and optimization (Lv et al., 2026).

### 6.3 Antimicrobial combination and antibiotic-adjuvant discovery

The continuous emergence of AMR has reduced the effectiveness of existing antibiotics, highlighting the need for alternative therapeutic strategies. Although the discovery of novel antimicrobial agents remains important, antibiotic combination therapy provides a promising strategy for combating AMR. However, experimentally identifying effective antibiotic combinations remains challenging because of the large number of possible combinations and the time-consuming and labor-intensive nature of experimental screening. Consequently, computational approaches have increasingly been investigated to facilitate antibiotic combination discovery and the identification of synergistic drug pairs (Lv et al., 2022a; Lv et al., 2022b; Lv et al., 2022c).

Lv et al. (2022a) proposed a graph learning framework for predicting synergistic antibiotic combinations. In this framework, network proximity combined with network propagation was used to quantify the relationships between drug pairs. The authors found that synergistic antibiotic combinations tended to have smaller network proximity. Network proximity was subsequently used to construct an affinity matrix, which was incorporated into a graph regularization model to predict potential synergistic antibiotic combinations. Compared with existing methods, the proposed framework showed better performance in predicting synergistic antibiotic combinations and provided interpretability for understanding drug-pair relationships.

The availability of appropriate data resources is important for computational antibiotic combination research. Lv et al. (2022b) developed the Antibiotic Combination DataBase (ACDB), which contains well-documented antibiotic combinations together with structural, physicochemical, pharmacological, and chemogenomic information. The database was developed to support AI-based models and the investigation of the mechanisms underlying synergy effects. In addition, Lv et al. (2022c) systematically reviewed computational models, databases, and tools for antibiotic combinations. The authors emphasized that computational methods have increasingly been used to predict effective antibiotic combinations, while several challenges remain, including the lack of large-scale and standardized datasets for antibiotic combinations. They also noted that existing models mainly focus on pairwise antibiotic combinations, with higher-order combinations involving three or more compounds receiving less attention. Moreover, combinations of antibiotics with antibiotic adjuvants have received relatively limited attention in existing computational studies.

Beyond direct prediction of antibiotic combinations, computational methods have also been applied to characterize drug–drug interaction (DDI) networks. Lv et al. (2023a) developed a similarity matrix fusion (SMF) method to integrate four types of drug information, including structural similarity, pharmaceutical similarity, phenotypic similarity, and therapeutic similarity. The fused similarity matrix was combined with t-distributed stochastic neighbor embedding (t-SNE) and hierarchical clustering to identify drug groups. The resulting group–group interactions were almost monochromatic. To evaluate clustering quality, the authors proposed edge purity and edge normalized mutual information. The clustered DDI network could also be used to predict new DDIs.

Drug similarity can also be measured directly from DDI networks. Lv et al. (2024) proposed a drug similarity measure based on DDI networks rather than conventional drug characteristics such as chemical structure and mechanism of action (MoA). When combined with unsupervised learning, this similarity measure enabled drugs to be grouped according to their interactions, and drugs within the same group tended to have similar MoAs. When combined with semi-supervised graph learning, the similarity measure could be used to construct affinity matrices and predict unknown DDIs. The authors suggested that this measure could be used for functional annotation of compounds with unknown MoA and prediction of unknown DDIs.

DDI information from multiple bacterial strains can further be used to identify antibiotic subgroups. Lv et al. (2023b) noted that previous studies had shown that antibiotics could be divided into groups and that DDI patterns depended on these groups, whereas previous analyses mainly focused on a specific bacterial strain, such as *Escherichia coli* BW25113. Because existing datasets often contain noise, the authors developed a multi-source information fusion method to integrate DDI information from multiple bacterial strains. Specifically, drug similarities were calculated from the DDI network of each bacterial strain and then fused into a new similarity matrix. The fused similarity matrix was combined with t-SNE and hierarchical clustering to identify antibiotic subgroups. These antibiotic subgroups were strongly correlated with known antibiotic classifications, and group–group interactions were almost monochromatic. The study therefore provided a framework for understanding the mechanisms of action of antibiotics and exploring multi-species group-group interactions.

Clustering methods have also been systematically reviewed for DDI network analysis. Cao et al. (2026) provided a comprehensive review of clustering methods for DDI networks, with emphasis on unsupervised approaches. The review introduced commonly used drug information and methods for calculating drug similarity, followed by representative clustering algorithms based on drug information and network information. The authors also discussed clustering evaluation metrics, limitations, and potential research directions. Compared with supervised learning approaches, clustering methods were highlighted for their potential in DDI network analysis and in uncovering mechanisms of action.

Antibiotic-adjuvant discovery represents another computational direction for combating AMR. Lv et al. (2023c) established the Antibiotic Adjuvant DataBase (AADB) by manually collecting relevant literature. AADB contains 3,035 combinations of antibiotics with adjuvants, covering 83 antibiotics, 226 adjuvants, and 325 bacterial strains. The database also includes related datasets, such as chemogenomic and metabolomic data, and provides a computational strategy for analyzing these datasets. As a test case, the authors identified 10 candidates for minocycline, of which 6 were known adjuvants that synergize with minocycline to inhibit the growth of *E. coli* BW25113. The authors suggested that AADB could help users identify effective antibiotic adjuvants.

Combining antibiotics with enzyme inhibitors represents an important antibiotic-adjuvant strategy for enhancing the activity of existing antibiotics against resistant bacteria. Shi et al. (2020) employed ML and DL models to discover seven potential New Delhi metallo-β-lactamase (NDM) inhibitors, providing a computational approach for identifying enzyme-targeting compounds that could be used in combination with antibiotics. However, owing to the limited availability of sample data, DL models did not demonstrate superior performance in the study. Bagdad et al. (2024) developed predictive models for noncovalent AmpC β-lactamase inhibitors using various ML models and a feedforward neural network (FFNN). The results showed that the FFNN achieved the best predictive performance among the tested models, demonstrating the potential of neural-network-based approaches for identifying antibiotic adjuvants targeting β-lactamase-mediated resistance.

LLMs have recently also been investigated for DDI network analysis. Chen et al. (2026) evaluated general LLMs, including ChatGPT5.2, DeepSeek3.2, and Gemini3.0, for DDI network clustering analysis. Using structured prompts incorporating drug information and DDI data, the authors found that LLMs could reconstruct known antibiotic classes when guided by drug information. When clustering was performed using only DDI interaction information, the models achieved high monochromaticity. The study suggested that LLMs can leverage domain knowledge embedded in their training corpora to support biomedical data analysis, while stability and reproducibility remain important considerations. Importantly, this study focused on DDI network clustering rather than directly predicting synergistic antibiotic combinations or antibiotic-adjuvant pairs.

Overall, computational studies of antimicrobial combinations and antibiotic adjuvants have covered several complementary tasks, including synergistic antibiotic combination prediction, DDI network analysis, drug similarity measurement, antibiotic subgroup identification, and antibiotic-adjuvant discovery. These studies have employed graph learning, graph regularization, network propagation, similarity matrix fusion, unsupervised learning, semi-supervised graph learning, hierarchical clustering, and LLM-based network analysis. However, several challenges remain, particularly the lack of large-scale and standardized antibiotic-combination datasets, the limited investigation of higher-order combinations involving three or more compounds, and the insufficient attention to antibiotic-adjuvant combinations. Further development of computational and deep learning approaches, together with expanded antibiotic-combination and antibiotic-adjuvant databases, may provide additional opportunities for identifying effective antimicrobial combinations and adjuvants for combating AMR.

### 6.4 Other antimicrobial drugs

In recent years, with the rising rates of AMR, research on bacteriophages has experienced a resurgence (Table 2). As a potential alternative therapy, bacteriophages are receiving increasing attention and are regarded as a promising solution for combating drug-resistant bacterial pathogens. Current tools for identifying phages and prophages primarily rely on sequence similarity, gene prediction, or the analysis of nucleotide k-mer distributions and specific sequence signatures (Auslander et al., 2020). However, most of these methods are only effective in detecting sequences that are highly similar to known viruses. Given the extremely high evolutionary rates of viral genomes, different viral populations often lack clear homology with previously identified viruses. Therefore, even using the most sensitive protein sequence alignment techniques, it remains challenging to accurately identify these highly variable viruses based on sequence similarity alone. Consequently, the effectiveness of existing identification tools is limited when dealing with rapidly evolving viruses. To address this challenge, researchers have begun exploring the application of DL techniques, achieving significant breakthroughs. Wei et al. (2024) developed a tool based on feature selection and DNN, DeepPBI-KG, for the prediction of phage-bacterial interactions. The results demonstrated that this method achieved favorable predictive performance, with an average area under the curve (AUC) of 0.93 across various strains, outperforming other existing prediction tools. Another study (Li M. et al., 2021) developed a tool based on CNN for predicting phage-host interactions (PredPHI), which is capable of identifying the host of phages from sequence data. Yang W. et al. (2024) proposed a DNN method based on the ESM-2 protein language model (SpikeHunter). They identified 231,965 distinct phage-encoded tailspike proteins in 787,566 bacterial genomes from 5 virulent, antibiotic-resistant pathogens. This study also found that phages with the same tailspike protein can infect different bacteria that possess similar polysaccharide receptors, further highlighting the central role of tailspike proteins in determining phage host range. Due to the fact that bacteriophages are among the most abundant biological entities on Earth and possess a rapid evolution rate, identifying unknown phages in sequence datasets remains a significant challenge. To address this issue, Auslander et al. (2020) presented an LSTM-based tool called Seeker for alignment-free identification of phage sequences. Seeker could efficiently detect phages from sequence datasets and accurately distinguish phages from sequences of bacterial origin, even if the sequence similarity between these phages and known phage families was extremely low. Fu et al. (2024) proposed a CNN model to identify phage lysins from the human microbiome. The authors found that the activity of the top candidate was 6.2-fold higher than that of lysozyme derived from egg white, making it the most effective lysin identified in the human microbiome to date.

Phage therapy represents an important alternative strategy for combating antimicrobial-resistant pathogens. However, compared with antibiotics and AMPs, the application of DL in the discovery of such antimicrobial drugs is still in its infancy. There remains considerable potential for further exploration and advancement in these fields.

## 7 Current challenges and future outlook

This review summarizes and discusses the significant progress made by DL technology in antibacterial research. DL technology, with its unique capabilities, can extract complex patterns and knowledge from extremely large amounts of data, which brings unprecedented opportunities to antibacterial research. Especially in predicting AMR mechanisms and discovering new antibacterial drugs, DL has demonstrated great potential and application prospects. By leveraging DL algorithms, researchers are better able to predict AMR with greater accuracy, thereby providing a scientific basis for the development of effective treatment strategies. At the same time, in the process of new drug development, DL accelerates the speed of screening candidate compounds with potential antimicrobial activity from large compound libraries, significantly shortening the research and development cycle and reducing costs. Although these achievements are encouraging, there are still some challenges that need to be considered in future research:

1. A major challenge in applying DL to antimicrobial research is the lack of standardized benchmark datasets for reliable model comparison. Although publicly available resources have greatly facilitated AMR prediction studies (Table 3), differences in data sources, resistance gene annotation, preprocessing strategies, and evaluation metrics limit the comparability of existing approaches. Models achieving high performance on specific datasets may show limited generalizability when applied to independent bacterial populations or emerging resistance determinants. Future efforts should prioritize the establishment of community-wide benchmark platforms integrating multiple AMR resources with standardized annotation schemes, quality control procedures, and predefined training, validation, and independent test sets. Unified evaluation protocols incorporating external validation and cross-species assessment will provide more objective evaluations of DL models and facilitate their practical application.
2. The complex architectures of DL models often hinder the interpretation of their predictions. In antimicrobial research, interpretability is particularly important because model-identified genomic or molecular features may provide insights into resistance mechanisms and guide antimicrobial discovery. Future studies should integrate explainable AI approaches with experimental validation to determine whether computationally identified features represent biologically meaningful mechanisms. Such efforts will enhance the reliability and applicability of DL-based antimicrobial approaches.
3. Many DL models are developed using relatively homogeneous datasets and may experience reduced performance when applied to unseen pathogens, novel resistance determinants, or external clinical datasets. Future research should focus on improving model generalizability through transfer learning, domain adaptation, and self-supervised learning strategies. These approaches may enable models to learn transferable biological representations from large-scale antimicrobial datasets and adapt to diverse antimicrobial tasks with limited labeled data. Evaluation using independent datasets from different bacterial species and geographic regions will be essential for assessing the robustness of DL models beyond their original training environments.
4. AMR is influenced by complex interactions among genomic variations, gene expression patterns, protein functions, molecular structures, and clinical factors. Therefore, future DL frameworks should integrate heterogeneous biological and clinical information through multimodal learning strategies rather than relying on single data modalities. In parallel, improving computational efficiency through optimized architectures and training strategies will facilitate practical deployment. Collaboration among microbiologists, clinicians, and AI researchers will be essential to ensure that DL-based predictions can be experimentally validated and translated into clinically meaningful applications.

**Table 3:** Representative data resources used in deep learning-based antimicrobial research.

Data resource	Application/task	Data modality	Data size	Accessibility	References
HMD-ARG-DB	ARG annotation and classification	Protein sequence	17,282 sequences	Publicly available	Li Y. et al. (2021)
ReSeqTB	*M. tuberculosis* resistance prediction	Short-read sequences	1,379 sequences	Publicly available	Chen et al. (2019)
DRIAMS	AMR prediction	Raw mass spectra	300,000 mass spectra	Publicly available	Weis et al. (2021)
PLSDB	Separating plasmids from bacterial chromosomes	Chromosomal and plasmid sequences	72,360 sequences	Publicly available	Molano et al. (2025)
DRAMP	Antimicrobial peptide prediction	Peptide sequence	30,260 Peptides	Publicly available	Kang et al. (2019)
ZINC15	Antibiotic discovery	Ligands	Over 120 million compound	Publicly available	Sterling and Irwin (2015)

## 8 Conclusion

This paper presents a comprehensive review of recent advances in the application of DL for predicting AMR mechanisms and discovering novel antimicrobial drugs, including antibiotics, AMPs, bacteriophages, and enzyme inhibitors. The review results show that, compared to traditional ML approaches, DL techniques do not rely on human-defined prior knowledge or complex feature engineering. Instead, they are capable of automatically extracting abstract features from large-scale and complex datasets, leading to significant achievements in the field of antimicrobial research. Moreover, these techniques have substantially streamlined the processes of predicting resistance mechanisms and discovering new antimicrobial drugs, overcoming the limitations of conventional methods such as high costs and long development cycles. In summary, the application of DL has ushered in a new era in antimicrobial research. By analyzing large-scale genomic data, researchers have not only gained deeper insights into the molecular mechanisms of drug resistance but also accelerated the drug discovery pipeline, offering strong support in addressing the global crisis of bacterial resistance. With the continuous advancement of AI technologies, it is expected that they will be more widely applied and explored in antibacterial research in the future. Furthermore, progress in these technologies will also promote interdisciplinary cooperation, such as integrating computational biology, chemoinformatics and other fields, to jointly drive the innovation and development of antibacterial treatment strategies and further address global public health challenges.

## References

[ref1] Ahmed N. Saha A. K. Al Noman M. A. Jim J. R. Mridha M. F. Kabir M. M. (2024). Deep learning-based natural language processing in human-agent interaction: applications, advancements and challenges. Nat. Lang. Proc. J. 9:100112. doi: 10.1016/j.nlp.2024.100112

[ref2] Akuthota U. C. Bhargava L. (2025). The role of machine and deep learning in modern intrusion detection systems: a comprehensive review. Comput. Electr. Eng. 124:110318. doi: 10.1016/j.compeleceng.2025.110318

[ref3] Algammal A. Hetta H. F. Mabrok M. Behzadi P. (2023). Editorial: emerging multidrug-resistant bacterial pathogens “superbugs”: A rising public health threat. Front. Microbiol. 14:1135614. doi: 10.3389/fmicb.2023.1135614, 36819057 PMC9930894

[ref4] Al-Selwi S. M. Hassan M. F. Abdulkadir S. J. Muneer A. Sumiea E. H. Alqushaibi A. . (2024). RNN-LSTM: from applications to modeling techniques and beyond-systematic review. J. King Saud Univ. 36:102068. doi: 10.1016/j.jksuci.2024.102068

[ref5] Aminov R. I. (2010). A brief history of the antibiotic era: lessons learned and challenges for the future. Front. Microbiol. 1:134. doi: 10.3389/fmicb.2010.00134, 21687759 PMC3109405

[ref6] Andreopoulos W. B. Geller A. M. Lucke M. Balewski J. Clum A. Ivanova N. N. . (2022). Deeplasmid: deep learning accurately separates plasmids from bacterial chromosomes. Nucleic Acids Res. 50:e17. doi: 10.1093/nar/gkab1115, 34871418 PMC8860608

[ref7] Arango-Argoty G. Garner E. Pruden A. Heath L. S. Vikesland P. Zhang L. (2018). DeepARG: a deep learning approach for predicting antibiotic resistance genes from metagenomic data. Microbiome 6:23. doi: 10.1186/s40168-018-0401-z, 29391044 PMC5796597

[ref8] Auslander N. Gussow A. B. Benler S. Wolf Y. I. Koonin E. V. (2020). Seeker: alignment-free identification of bacteriophage genomes by deep learning. Nucleic Acids Res. 48:e121. doi: 10.1093/nar/gkaa856, 33045744 PMC7708075

[ref9] Bagdad Y. Sisquellas M. Arthur M. Miteva M. A. (2024). Machine learning and deep learning models for predicting noncovalent inhibitors of AmpC beta-lactamase. ACS Omega 9, 41334–41342. doi: 10.1021/acsomega.4c03834, 39398163 PMC11465252

[ref11] Baquero F. Martinez J. L. Lanza V. F. Rodriguez-Beltran J. Galan J. C. San Millan A. . (2021). Evolutionary pathways and trajectories in antibiotic resistance. Clin. Microbiol. Rev. 34:e0005019. doi: 10.1128/cmr.00050-19, 34190572 PMC8404696

[ref12] Baranova A. A. Alferova V. A. Korshun V. A. Tyurin A. P. (2023). Modern trends in natural antibiotic discovery. Life (Basel) 13:1073. doi: 10.3390/life13051073, 37240718 PMC10221674

[ref13] BD (2025). Antimicrobial Resistance: Uncovering the Challenges and Key Strategies for Action BD. Franklin Lakes, NJ, USA: BD Report.

[ref14] Bentley D. R. Balasubramanian S. Swerdlow H. P. Smith G. P. Milton J. Brown C. G. . (2008). Accurate whole human genome sequencing using reversible terminator chemistry. Nature 456, 53–59. doi: 10.1038/nature07517, 18987734 PMC2581791

[ref15] Bilal H. Khan M. N. Khan S. Shafiq M. Fang W. Khan R. U. . (2025). The role of artificial intelligence and machine learning in predicting and combating antimicrobial resistance. Comput. Struct. Biotechnol. J. 27, 423–439. doi: 10.1016/j.csbj.2025.01.006, 39906157 PMC11791014

[ref16] Boulaamane Y. Panadero I. M. Hmadcha A. Rey C. A. Baammi S. Allali A. E. . (2024). Antibiotic discovery with artificial intelligence for the treatment of *Acinetobacter baumannii* infections. mSystems 9:e0032524. doi: 10.1128/msystems.00325-24, 38700330 PMC11326114

[ref18] Branton D. Deamer D. W. Marziali A. Bayley H. Benner S. A. Butler T. . (2008). The potential and challenges of nanopore sequencing. Nat. Biotechnol. 26, 1146–1153. doi: 10.1038/nbt.1495, 18846088 PMC2683588

[ref19] Brown T. Mann B. Ryder N. Subbiah M. Kaplan J. Dhariwal P. . (2020). “Language models are few-shot learners,” in 34th Conference on Neural Information Processing Systems (NeurIPS), (Red Hook, NY, USA: Curran Associates, Inc.), 33.

[ref20] Cao S. Liu G. Zhou X. Lv J. (2026). A comprehensive review of cluster methods for drug–drug interaction network. Quant. Biol.:e70015. doi: 10.1002/qub2.70015 PMC12806105 41676326

[ref21] Cesaro A. Bagheri M. Torres M. Wan F. de la Fuente-Nunez C. (2023). Deep learning tools to accelerate antibiotic discovery. Expert Opin. Drug Discov. 18, 1245–1257. doi: 10.1080/17460441.2023.2250721, 37794737 PMC10790350

[ref22] Chain E. Florey H. W. Adelaide M. B. Gardner A. D. Heatley N. G. Jennings M. A. . (2005). The classic: penicillin as a chemotherapeutic agent. Clin. Orthop. Relat. Res. 295, 3–7. doi: 10.1097/01.blo.0000183429.83168.07 8403666

[ref23] Chen M. L. Doddi A. Royer J. Freschi L. Schito M. Ezewudo M. . (2019). Beyond multidrug resistance: leveraging rare variants with machine and statistical learning models in *Mycobacterium tuberculosis* resistance prediction. EBioMedicine 43, 356–369. doi: 10.1016/j.ebiom.2019.04.016, 31047860 PMC6557804

[ref24] Chen Y. Liu G. Lv J. (2026). Evaluating the ability of large language models in drug-drug interaction network clustering analysis. Curr. Bioinforma. 21. doi: 10.2174/0115748936454145260511200213

[ref25] Chowdhery A. Narang S. Devlin J. Bosma M. Mishra G. Roberts A. . (2023). PaLM: scaling language modeling with pathways. J. Mach. Learn. Res. 24:240. doi: 10.48550/arXiv.2204.02311

[ref27] Cook M. A. Wright G. D. (2022). The past, present, and future of antibiotics. Sci. Transl. Med. 14:eabo7793. doi: 10.1126/scitranslmed.abo7793, 35947678

[ref28] Davies D. T. Everett M. (2021). Designing inhibitors of beta-lactamase enzymes to overcome carbapenem resistance in gram-negative bacteria. Acc. Chem. Res. 54, 2055–2064. doi: 10.1021/acs.accounts.0c00863, 33788541

[ref29] Djordjevic S. P. Jarocki V. M. Seemann T. Cummins M. L. Watt A. E. Drigo B. . (2024). Genomic surveillance for antimicrobial resistance – a one health perspective. Nat. Rev. Genet. 25, 142–157. doi: 10.1038/s41576-023-00649-y, 37749210

[ref30] Dong Y. Quan H. Ma C. Shan L. Deng L. (2024). TGC-ARG: anticipating antibiotic resistance via transformer-based modeling and contrastive learning. Int. J. Mol. Sci. 25:7228. doi: 10.3390/ijms25137228, 39000335 PMC11241484

[ref31] Durand G. A. Raoult D. Dubourg G. (2019). Antibiotic discovery: history, methods and perspectives. Int. J. Antimicrob. Agents 53, 371–382. doi: 10.1016/j.ijantimicag.2018.11.010, 30472287

[ref32] Eid J. Fehr A. Gray J. Luong K. Lyle J. Otto G. . (2009). Real-time DNA sequencing from single polymerase molecules. Science 323, 133–138. doi: 10.1126/science.1162986, 19023044

[ref33] El Haddad L. Harb C. P. Gebara M. A. Stibich M. A. Chemaly R. F. (2019). A systematic and critical review of bacteriophage therapy against multidrug-resistant ESKAPE organisms in humans. Clin. Infect. Dis. 69, 167–178. doi: 10.1093/cid/ciy947, 30395179

[ref34] Elnaggar A. Heinzinger M. Dallago C. Rehawi G. Wang Y. Jones L. . (2022). ProtTrans: toward understanding the language of life through self-supervised learning. IEEE Trans. Pattern Anal. Mach. Intell. 44, 7112–7127. doi: 10.1109/TPAMI.2021.3095381, 34232869

[ref35] Fleischmann R. D. Adams M. D. White O. Clayton R. A. Kirkness E. F. Kerlavage A. R. . (1995). Whole-genome random sequencing and assembly of *Haemophilus influenzae* Rd. Science 269, 496–512. doi: 10.1126/science.7542800, 7542800

[ref36] Fu H. Cao Z. Li M. Wang S. (2020). ACEP: improving antimicrobial peptides recognition through automatic feature fusion and amino acid embedding. BMC Genomics 21:597. doi: 10.1186/s12864-020-06978-0, 32859150 PMC7455913

[ref37] Fu Y. Yu S. Li J. Lao Z. Yang X. Lin Z. (2024). DeepMineLys: deep mining of phage lysins from human microbiome. Cell Rep. 43:114583. doi: 10.1016/j.celrep.2024.114583, 39110597

[ref38] Gandhi N. R. Nunn P. Dheda K. Schaaf H. S. Zignol M. van Soolingen D. . (2010). Multidrug-resistant and extensively drug-resistant tuberculosis: a threat to global control of tuberculosis. Lancet 375, 1830–1843. doi: 10.1016/S0140-6736(10)60410-2, 20488523

[ref40] Gao W. Li H. Yang J. Zhang J. Fu R. Peng J. . (2024). Machine learning assisted MALDI mass spectrometry for rapid antimicrobial resistance prediction in clinicals. Anal. Chem. 96, 13398–13409. doi: 10.1021/acs.analchem.4c00741, 39096240

[ref41] Goodfellow I. J. Pouget-Abadie J. Mirza M. Xu B. Warde-Farley D. Ozair S. . (2014). Generative adversarial networks. Adv. Neural Informat. Proc. Syst. 27, 2672–2680.

[ref42] Guan C. Fernandes F. C. Franco O. L. de la Fuente-Nunez C. (2025). Leveraging large language models for peptide antibiotic design. Cell Rep. Phys. Sci. 6:102359. doi: 10.1016/j.xcrp.2024.102359, 39949833 PMC11823563

[ref43] Hamid M. N. Friedberg I. (2019). Identifying antimicrobial peptides using word embedding with deep recurrent neural networks. Bioinformatics 35, 2009–2016. doi: 10.1093/bioinformatics/bty937, 30418485 PMC6581433

[ref4001] Hancock R. E. W. Alford M. A. Haney E. F. (2021). Antibiofilm activity of host defence peptides: complexity provides opportunities. Nat Rev Microbiol 19, 786–797. doi: 10.1038/s41579-021-00585-w, 34183822

[ref44] Hao Y. Liu X. Fu H. Shao X. Cai W. (2024). PGAT-ABPp: harnessing protein language models and graph attention networks for antibacterial peptide identification with remarkable accuracy. Bioinformatics 40:btae497. doi: 10.1093/bioinformatics/btae497, 39120878 PMC11338452

[ref45] He S. Ye X. Dou L. Sakurai T. (2024). FIAMol-AB: A feature fusion and attention-based deep learning method for enhanced antibiotic discovery. Comput. Biol. Med. 168:107762. doi: 10.1016/j.compbiomed.2023.107762, 38056212

[ref47] Ho J. Jain A. Abbeel P. (2020). Denoising diffusion probabilistic models. Adv. Neural Informat. Proc. Syst. 33, 6840–6851. doi: 10.48550/arXiv.2006.11239

[ref48] Ho C. S. Wong C. T. H. Aung T. T. Lakshminarayanan R. Mehta J. S. Rauz S. . (2025). Antimicrobial resistance: a concise update. Lancet Microbe 6:100947. doi: 10.1016/j.lanmic.2024.07.010, 39305919

[ref49] Iskandar K. Murugaiyan J. Hammoudi Halat D. Hage S. E. Chibabhai V. Adukkadukkam S. . (2022). Antibiotic discovery and resistance: the chase and the race. Antibiotics (Basel) 11:182. doi: 10.3390/antibiotics11020182, 35203785 PMC8868473

[ref50] Ji Y. Zhou Z. Liu H. Davuluri R. V. (2021). DNABERT: pre-trained bidirectional encoder representations from transformers model for DNA-language in genome. Bioinformatics 37, 2112–2120. doi: 10.1093/bioinformatics/btab083, 33538820 PMC11025658

[ref51] Jia H. Li X. Zhuang Y. Wu Y. Shi S. Sun Q. . (2024). Neural network-based predictions of antimicrobial resistance phenotypes in multidrug-resistant *Acinetobacter baumannii* from whole genome sequencing and gene expression. Antimicrob. Agents Chemother. 68, e01446–e01424. doi: 10.1128/aac.01446-24, 39540735 PMC11619347

[ref52] Jiang Z. Lu Y. Liu Z. Wu W. Xu X. Dinnyes A. . (2022). Drug resistance prediction and resistance genes identification in *Mycobacterium tuberculosis* based on a hierarchical attentive neural network utilizing genome-wide variants. Brief. Bioinform. 23:bbac041. doi: 10.1093/bib/bbac041, 35325021

[ref53] Jumper J. Evans R. Pritzel A. Green T. Figurnov M. Ronneberger O. . (2021). Highly accurate protein structure prediction with AlphaFold. Nature 596, 583–589. doi: 10.1038/s41586-021-03819-2, 34265844 PMC8371605

[ref54] Kang X. Dong F. Shi C. Liu S. Sun J. Chen J. . (2019). DRAMP 2.0, an updated data repository of antimicrobial peptides. Sci. Data 6:148. doi: 10.1038/s41597-019-0154-y, 31409791 PMC6692298

[ref55] Kayama S. Yahara K. Sugawara Y. Kawakami S. Kondo K. Zuo H. . (2023). National genomic surveillance integrating standardized quantitative susceptibility testing clarifies antimicrobial resistance in Enterobacterales. Nat. Commun. 14:8046. doi: 10.1038/s41467-023-43516-4, 38052776 PMC10698200

[ref56] Khaledi A. Weimann A. Schniederjans M. Asgari E. Kuo T. H. Oliver A. . (2020). Predicting antimicrobial resistance in *Pseudomonas aeruginosa* with machine learning-enabled molecular diagnostics. EMBO Mol. Med. 12:e10264. doi: 10.15252/emmm.201910264, 32048461 PMC7059009

[ref57] Khedkar S. Smyshlyaev G. Letunic I. Maistrenko O. M. Coelho L. P. Orakov A. . (2022). Landscape of mobile genetic elements and their antibiotic resistance cargo in prokaryotic genomes. Nucleic Acids Res. 50, 3155–3168. doi: 10.1093/nar/gkac163, 35323968 PMC8989519

[ref58] Kingma D. P. Welling M. (2014). Auto-Encoding Variational Bayes International Conference on Learning Representations (ICLR). Ithaca, NY, USA: arXiv.

[ref59] Koubaa A. (2023). GPT-4 vs. GPT-3.5: a concise showdown. Preprints:2023030422. doi: 10.36227/techrxiv.22312330.v2

[ref60] Kromer-Edwards C. Castanheira M. Oliveira S. (2022). K-Mer Fingerprinting with RNN to Predict MICs for *K. pneumoniae*. 2022 IEEE International Conference on Bioinformatics and Biomedicine (BIBM). Piscataway, NJ, USA: IEEE.

[ref61] Kwon H. C. Kauffman C. A. Jensen P. R. Fenical W. (2006). Marinomycins A-D, antitumor-antibiotics of a new structure class from a marine actinomycete of the recently discovered genus “marinispora”. J. Am. Chem. Soc. 128, 1622–1632. doi: 10.1021/ja0558948, 16448135

[ref62] Kwon J. H. Powderly W. G. (2021). The post-antibiotic era is here. Science 373:471. doi: 10.1126/science.abl5997, 34326211

[ref63] LeCun Y. Bengio Y. Hinton G. (2015). Deep learning. Nature 521, 436–444. doi: 10.1038/nature14539 26017442

[ref64] LeCun Y. Bottou L. Bengio Y. Haffner P. (1998). Gradient-based learning applied to document recognition. Proc. IEEE 86, 2278–2324. doi: 10.1109/5.726791

[ref66] Levine D. P. (2006). Vancomycin: a history. Clin. Infect. Dis. 42, S5–S12. doi: 10.1086/491709 16323120

[ref67] Lewis K. (2012). Antibiotics: recover the lost art of drug discovery. Nature 485, 439–440. doi: 10.1038/485439a 22622552

[ref68] Lewis K. (2013). Platforms for antibiotic discovery. Nat. Rev. Drug Discov. 12, 371–387. doi: 10.1038/nrd3975, 23629505

[ref69] Li C. Sutherland D. Richter A. Coombe L. Yanai A. Warren R. L. . (2024). De novo synthetic antimicrobial peptide design with a recurrent neural network. Protein Sci. 33:e5088. doi: 10.1002/pro.5088, 38988311 PMC11237553

[ref70] Li M. Wang Y. Li F. Zhao Y. Liu M. Zhang S. . (2021). A deep learning-based method for identification of bacteriophage-host interaction. IEEE/ACM Trans. Comput. Biol. Bioinform. 18, 1801–1810. doi: 10.1109/tcbb.2020.3017386, 32813660 PMC8703204

[ref71] Li Y. Xu Z. Han W. Cao H. Umarov R. Yan A. . (2021). HMD-ARG: hierarchical multi-task deep learning for annotating antibiotic resistance genes. Microbiome 9:40. doi: 10.1186/s40168-021-01002-3, 33557954 PMC7871585

[ref72] Lin Z. Akin H. Rao R. Hie B. Zhu Z. Lu W. . (2023). Evolutionary-scale prediction of atomic-level protein structure with a language model. Science 379, 1123–1130. doi: 10.1126/science.ade2574 36927031

[ref73] Lin B. Yan S. Zhen B. (2025). A machine learning method for predicting molecular antimicrobial activity. Sci. Rep. 15:6559. doi: 10.1038/s41598-025-91190-x, 39994442 PMC11850884

[ref74] Lindsay J. A. (2014). *Staphylococcus aureus* genomics and the impact of horizontal gene transfer. Int. J. Med. Microbiol. 304, 103–109. doi: 10.1016/j.ijmm.2013.11.010, 24439196

[ref75] Liu G. Catacutan D. B. Rathod K. Swanson K. Jin W. Mohammed J. C. . (2023). Deep learning-guided discovery of an antibiotic targeting *Acinetobacter baumannii*. Nat. Chem. Biol. 19, 1342–1350. doi: 10.1038/s41589-023-01349-8 37231267

[ref76] Lluka T. Stokes J. M. (2023). Antibiotic discovery in the artificial intelligence era. Ann. N. Y. Acad. Sci. 1519, 74–93. doi: 10.1111/nyas.14930 36447334

[ref77] Logan L. K. Weinstein R. A. (2017). The epidemiology of carbapenem-resistant Enterobacteriaceae: the impact and evolution of a global menace. J. Infect. Dis. 215, S28–S36. doi: 10.1093/infdis/jiw282, 28375512 PMC5853342

[ref78] Lopez-Cortes X. A. Manriquez-Troncoso J. M. Hernandez-Garcia R. Peralta D. (2024). MSDeepAMR: antimicrobial resistance prediction based on deep neural networks and transfer learning. Front. Microbiol. 15:1361795. doi: 10.3389/fmicb.2024.1361795, 38694798 PMC11062410

[ref79] Lv J. Liu G. Dong W. Ju Y. Sun Y. (2022b). ACDB: an antibiotic combination DataBase. Front. Pharmacol. 13:869983. doi: 10.3389/fphar.2022.869983, 35370670 PMC8971807

[ref80] Lv J. Liu G. Hao J. Ju Y. Sun B. Sun Y. (2022c). Computational models, databases and tools for antibiotic combinations. Brief. Bioinform. 23, 1–11. doi: 10.1093/bib/bbac309, 35915052

[ref81] Lv J. Liu G. Ju Y. Huang H. Li D. Sun Y. (2023b). Identification of robust antibiotic subgroups by integrating multi-species drug–drug interactions. J. Chem. Inf. Model. 63, 4970–4978. doi: 10.1021/acs.jcim.3c00937 37459588

[ref82] Lv J. Liu G. Ju Y. Huang H. Sun Y. (2023c). AADB: A manually collected database for combinations of antibiotics with adjuvants. IEEE/ACM Trans. Comput. Biol. Bioinform. 20, 2827–2836. doi: 10.1109/TCBB.2023.3283221, 37279138

[ref83] Lv J. Liu G. Ju Y. Huang H. Sun Y. (2024). Measuring drug similarity using drug–drug interactions. Quant. Biol. 12, 164–172. doi: 10.1002/qub2.38, 41676374 PMC12806202

[ref84] Lv J. Liu G. Ju Y. Sun Y. Guo W. (2022a). Prediction of synergistic antibiotic combinations by graph learning. Front. Pharmacol. 13:849006. doi: 10.3389/fphar.2022.849006, 35350764 PMC8958015

[ref85] Lv J. Liu G. Ju Y. Sun B. Huang H. Sun Y. (2023a). Integrating multi-source drug information to cluster drug-drug interaction network. Comput. Biol. Med. 162:107088. doi: 10.1016/j.compbiomed.2023.107088, 37263154

[ref86] Lv J. Liu G. Zhou X. Chen Y. (2026). Artificial intelligence-driven antimicrobial peptide discovery: prediction, generation, mining and optimization. Probiotics Antimicrob. Proteins 18, 9157–9171. doi: 10.1007/s12602-026-11013-x, 42020672

[ref87] Margulies M. Egholm M. Altman W. E. Attiya S. Bader J. S. Bemben L. A. . (2005). Genome sequencing in microfabricated high-density picolitre reactors. Nature 437, 376–380. doi: 10.1038/nature03959, 16056220 PMC1464427

[ref88] Maxam A. M. Gilbert W. (1977). New method for sequencing DNA. P Natl Acad SCI USA 74, 560–564. doi: 10.1073/pnas.74.2.560, 265521 PMC392330

[ref89] Melo M. C. R. Maasch J. R. M. A. de la Fuente-Nunez C. (2021). Accelerating antibiotic discovery through artificial intelligence. Commun. Biol. 4:1050. doi: 10.1038/s42003-021-02586-0, 34504303 PMC8429579

[ref90] Molano L. A. G. Hirsch P. Hannig M. Molano L. G. Müller R. Keller A. (2025). The PLSDB 2025 update: enhanced annotations and improved functionality for comprehensive plasmid research. Nucleic Acids Res. 53, D189–D196. doi: 10.1093/nar/gkae1095, 39565221 PMC11701622

[ref91] Naghavi M. Vollset S. E. Ikuta K. S. Swetschinski L. R. Gray A. P. Wool E. E. . (2024). Global burden of bacterial antimicrobial resistance 1990-2021: a systematic analysis with forecasts to 2050. Lancet 404, 1199–1226. doi: 10.1016/S0140-6736(24)01867-1, 39299261 PMC11718157

[ref92] Nguyen M. Long S. W. McDermott P. F. Olsen R. J. Olson R. Stevens R. L. . (2019). Using machine learning to predict antimicrobial MICs and associated genomic features for nontyphoidal *Salmonella*. J. Clin. Microbiol. 57:e01260-18. doi: 10.1128/jcm.01260-18, 30333126 PMC6355527

[ref93] Nguyen H. A. Peleg A. Y. Song J. Antony B. Webb G. I. Wisniewski J. A. . (2024). Predicting *Pseudomonas aeruginosa* drug resistance using artificial intelligence and clinical MALDI-TOF mass spectra. mSystems 9, e00789–e00724. doi: 10.1128/msystems.00789-24, 39150244 PMC11406958

[ref95] Nordmann P. Yao Y. Falgenhauer L. Sadek M. Imirzalioglu C. Chakraborty T. (2021). Recent emergence of aztreonam-avibactam resistance in NDM and OXA-48 Carbapenemase-producing *Escherichia coli* in Germany. Antimicrob. Agents Chemother. 65:e0109021. doi: 10.1128/AAC.01090-21, 34424048 PMC8522741

[ref96] O’Neill J. (2016). Tackling Drug-Resistant Infections Globally: Final Report and Recommendations. London, UK: Review on Antimicrobial Resistance.

[ref97] Okeke I. N. de Kraker M. E. A. Van Boeckel T. P. Kumar C. K. Schmitt H. Gales A. C. . (2024). The scope of the antimicrobial resistance challenge. Lancet 403, 2426–2438. doi: 10.1016/s0140-6736(24)00876-6, 38797176

[ref98] Ong'uti S. Czech M. Robilotti E. Holubar M. (2022). Cefiderocol: a new cephalosporin stratagem against multidrug-resistant gram-negative bacteria. Clin. Infect. Dis. 74, 1303–1312. doi: 10.1093/cid/ciab757, 34492098 PMC9989360

[ref99] Pang Y. Yao L. Xu J. Wang Z. Lee T. Y. (2022). Integrating transformer and imbalanced multi-label learning to identify antimicrobial peptides and their functional activities. Bioinformatics 38, 5368–5374. doi: 10.1093/bioinformatics/btac711, 36326438 PMC9750108

[ref100] Pei Y. Shum M. H. Liao Y. Leung V. W. Gong Y. N. Smith D. K. . (2024). ARGNet: using deep neural networks for robust identification and classification of antibiotic resistance genes from sequences. Microbiome 12:84. doi: 10.1186/s40168-024-01805-0, 38725076 PMC11080312

[ref101] Ribeiro da Cunha B. Fonseca L. P. Calado C. R. C. (2019). Antibiotic discovery: where have we come from, where do we go? Antibiotics (Basel) 8:45. doi: 10.3390/antibiotics8020045, 31022923 PMC6627412

[ref103] Sanger F. Nicklen S. Coulson A. R. (1977). DNA sequencing with chain-terminating inhibitors. Proc. Natl. Acad. Sci. 74, 5463–5467. doi: 10.1073/pnas.74.12.5463, 271968 PMC431765

[ref104] Scarselli F. Gori M. Tsoi A. C. Hagenbuchner M. Monfardini G. (2009). The graph neural network model. IEEE T. Neural Netw. 20, 61–80. doi: 10.1109/TNN.2008.2005605 19068426

[ref105] Shendure J. Porreca G. J. Reppas N. B. Lin X. McCutcheon J. P. Rosenbaum A. M. . (2005). Accurate multiplex polony sequencing of an evolved bacterial genome. Science 309, 1728–1732. doi: 10.1126/science.1117389, 16081699

[ref106] Shi C. Dong F. Zhao G. Zhu N. Lao X. Zhang H. (2020). Applications of machine-learning methods for the discovery of NDM-1 inhibitors. Chem. Biol. Drug Des. 96, 1232–1243. doi: 10.1111/cbdd.13708, 32418370

[ref107] Silva M. K. P. Nicoleti V. Y. U. Rodrigues B. Araujo A. S. F. Ellwanger J. H. de Almeida J. M. . (2025). Exploring deep learning in phage discovery and characterization. Virology 609:110559. doi: 10.1016/j.virol.2025.110559 40359589

[ref108] Smith L. M. Sanders J. Z. Kaiser R. J. Hughes P. Dodd C. Connell C. R. . (1986). Fluorescence detection in automated DNA sequence analysis. Nature 321, 674–679. doi: 10.1038/321674a0, 3713851

[ref109] Sterling T. Irwin J. J. (2015). ZINC 15–ligand discovery for everyone. J. Chem. Inf. Model. 55, 2324–2337. doi: 10.1021/acs.jcim.5b00559, 26479676 PMC4658288

[ref110] Stewardson A. J. Marimuthu K. Sengupta S. Allignol A. el-Bouseary M. Carvalho M. J. . (2019). Effect of carbapenem resistance on outcomes of bloodstream infection caused by Enterobacteriaceae in low-income and middle-income countries (PANORAMA): a multinational prospective cohort study. Lancet Infect. Dis. 19, 601–610. doi: 10.1016/S1473-3099(18)30792-8, 31047852

[ref111] Stokes J. M. Yang K. Swanson K. Jin W. Cubillos-Ruiz A. Donghia N. M. . (2020). A deep learning approach to antibiotic discovery. Cell 180, 688–702.e13. doi: 10.1016/j.cell.2020.01.021, 32084340 PMC8349178

[ref112] Tang X. Shang J. Ji Y. Sun Y. (2023). PLASMe: a tool to identify PLASMid contigs from short-read assemblies using transformer. Nucleic Acids Res. 51:e83. doi: 10.1093/nar/gkad578, 37427782 PMC10450166

[ref113] The Pew Charitable Trusts (2021). Tracking the Global Pipeline of Antibiotics in Development. Washington, DC: The Pew Charitable.

[ref114] Touvron H. Lavril T. Izacard G. Martinet X. Lachaux M. Lacroix T. . (2023). LLaMA: open and efficient foundation language models. arXiv:2302.13971. doi: 10.48550/arXiv.2302.13971

[ref115] Van Camp P. J. Haslam D. B. Porollo A. (2020). Prediction of antimicrobial resistance in gram-negative bacteria from whole-genome sequencing data. Front. Microbiol. 11:1013. doi: 10.3389/fmicb.2020.01013, 32528441 PMC7262952

[ref116] Vaswani A. Shazeer N. Parmar N. Uszkoreit J. Jones L. Gomez A. N. . (2017). Attention Is All You Need Advances in Neural Information Processing Systems, (Red Hook, NY, USA: Curran Associates, Inc.), 30.

[ref117] Vuong C. Yeh A. J. Cheung G. Y. Otto M. (2016). Investigational drugs to treat methicillin-resistant *Staphylococcus aureus*. Expert Opin. Investig. Drugs 25, 73–93. doi: 10.1517/13543784.2016.1109077, 26536498 PMC5004000

[ref118] Walker T. M. Clp C. L. Harrell R. H. Cummins M. L. Watt A. E. Drigo B. . (2013). Whole-genome sequencing to delineate *Mycobacterium tuberculosis* outbreaks: a retrospective observational study. Lancet Infect. Dis. 13, 137–146. doi: 10.1016/S1473-3099(12)70277-3 PMC3556524 23158499

[ref119] Walker T. M. Kohl T. A. Omar S. V. Evans J. T. Kapatai G. Dedicoat M. J. . (2015). Whole-genome sequencing for prediction of *Mycobacterium tuberculosis* drug susceptibility and resistance: a retrospective cohort study. Lancet Infect. Dis. 15, 1193–1202. doi: 10.1016/S1473-3099(15)00062-6, 26116186 PMC4579482

[ref120] Wang X. Deng Z. Gao J. (2024). Exploring the antibiotic potential of cultured 'unculturable' bacteria. Trends Microbiol. 32, 124–127. doi: 10.1016/j.tim.2023.11.012, 38102034

[ref121] Wang C. Garlick S. Zloh M. (2021). Deep learning for novel antimicrobial peptide design. Biomolecules 11:471. doi: 10.3390/biom11030471, 33810011 PMC8004669

[ref122] Wang Y. Zheng S. Guo R. Li Y. Yin H. Qiu X. . (2025). Assessment for antibiotic resistance in *Helicobacter pylori*: a practical and interpretable machine learning model based on genome-wide genetic variation. Virulence 16:2481503. doi: 10.1080/21505594.2025.2481503, 40119500 PMC11934168

[ref123] Waqas M. Humphries U. W. (2024). A critical review of RNN and LSTM variants in hydrological time series predictions. MethodsX 13:102946. doi: 10.1016/j.mex.2024.102946, 39324077 PMC11422155

[ref124] Waqas M. Naseem A. Humphries U. W. Hlaing P. T. Dechpichai P. Wangwongchai A. (2025). Applications of machine learning and deep learning in agriculture: a comprehensive review. Green Technol. Sustainability 3:100199. doi: 10.1016/j.grets.2025.100199

[ref125] Watson J. D. Crick F. H. (1953). Molecular structure of nucleic acids: a structure for deoxyribose nucleic acid. Nature 171, 737–738. doi: 10.1038/171737a0, 13054692

[ref126] Wei T. Lu C. Du H. Yang Q. Qi X. Liu Y. . (2024). DeepPBI-KG: a deep learning method for the prediction of phage-bacteria interactions based on key genes. Brief. Bioinform. 25:bbae484. doi: 10.1093/bib/bbae484, 39344712 PMC11440089

[ref127] Weis C. Cuénod A. Rieck B. Borgwardt K. Egli A. (2021). DRIAMS: Database of Resistance Information on Antimicrobials and MALDI-TOF Mass Spectra. California, USA: Dryad, Dataset.

[ref128] Wheeler N. E. Price V. Cunningham-Oakes E. Tsang K. K. Nunn J. G. Midega J. T. . (2023). Innovations in genomic antimicrobial resistance surveillance. Lancet Microbe 4, E1063–E1070. doi: 10.1016/S2666-5247(23)00285-9, 37977163

[ref129] Wong F. Zheng E. J. Valeri J. A. Donghia N. M. Anahtar M. N. Omori S. . (2024). Discovery of a structural class of antibiotics with explainable deep learning. Nature 626, 177–185. doi: 10.1038/s41586-023-06887-8, 38123686 PMC10866013

[ref130] Wu J. Ouyang J. Qin H. Zhou J. Roberts R. Siam R. . (2023). PLM-ARG: antibiotic resistance gene identification using a pretrained protein language model. Bioinformatics 39:btad690. doi: 10.1093/bioinformatics/btad690, 37995287 PMC10676515

[ref131] Xia X. Zhu C. Zhong F. Liu L. (2023). MDTips: a multimodal-data-based drug-target interaction prediction system fusing knowledge, gene expression profile, and structural data. Bioinformatics 39:btad411. doi: 10.1093/bioinformatics/btad411, 37379157 PMC10329491

[ref132] Xu J. Li F. Li C. Guo X. Landersdorfer C. Shen H. H. . (2023). iAMPCN: a deep-learning approach for identifying antimicrobial peptides and their functional activities. Brief. Bioinform. 24, 1–20. doi: 10.1093/bib/bbad240, 37369638 PMC10359087

[ref135] Yang Y. Dufault-Thompson K. Yan W. Cai T. Xie L. Jiang X. (2024). Large-scale genomic survey with deep learning-based method reveals strain-level phage specificity determinants. Gigascience 13:giae017. doi: 10.1093/gigascience/giae017, 38649301 PMC11034027

[ref136] Yang W. Li L. Bai S. Ma Z. (2024). IS-GNN: graph neural network enhanced by aggregating influential and structurally similar nodes. Knowl. Based Syst. 301:112282. doi: 10.1016/j.knosys.2024.112282

[ref137] Yang Y. Walker T. M. Walker A. S. Wilson D. J. Peto T. E. A. Crook D. W. . (2019). DeepAMR for predicting co-occurrent resistance of *Mycobacterium tuberculosis*. Bioinformatics 35, 3240–3249. doi: 10.1093/bioinformatics/btz067, 30689732 PMC6748723

[ref138] Yang C. L. Wang P. P. Zhou Z. Y. Wu X. W. Hua Y. Chen J. W. . (2025). Discovery of naturally inspired antimicrobial peptides using deep learning. Bioorg. Chem. 160:108444. doi: 10.1016/j.bioorg.2025.108444, 40209356

[ref139] Zhao W. Wu J. Jiang X. He T. Hu X. (2024). Causal-ARG- a causality-guided framework for annotating properties of antibiotic resistance genes. Bioinformatics 40:btae180. doi: 10.1093/bioinformatics/btae180, 38569882 PMC11026140

[ref140] Zheng X. Zhang L. Xu C. Chen X. Cui Z. (2024). An attribution graph-based interpretable method for CNNs. Neural Netw. 179:106597. doi: 10.1016/j.neunet.2024.106597, 39128275

[ref141] Zhou H. Beltran J. F. Brito I. L. (2021). Functions predict horizontal gene transfer and the emergence of antibiotic resistance. Sci. Adv. 7:eabj5056. doi: 10.1126/sciadv.abj5056, 34678056 PMC8535800

[ref142] Zhou J. Cui G. Zhang Z. Hu S. Yang C. Liu Z. . (2020). Graph neural networks: a review of methods and applications. AI Open 1, 57–81. doi: 10.1016/j.aiopen.2021.01.001

[ref143] Zhou X. Liu G. Cao S. Lv J. (2025a). Deep learning for antimicrobial peptides: computational models and databases. J. Chem. Inf. Model. 65, 1708–1717. doi: 10.1021/acs.jcim.5c00006 39927895

[ref144] Zhou X. Liu G. Han C. Lv J. (2025b). ABPDB: A database of antibacterial peptides. IEEE Trans Comput Biol Bioinform 22, 2300–2306. doi: 10.1109/TCBBIO.2025.3582844, 40811252

[ref145] Zhou X. Liu G. Lv J. (2026). CLABP: a contrastive learning framework integrating protein language models and structural information for antibacterial peptide prediction. Brief. Bioinform. 27:bbag335. doi: 10.1093/bib/bbag335, 42366619 PMC13310757

